# Deubiquitinase catalytic activity of MYSM1 is essential in vivo for hematopoiesis and immune cell development

**DOI:** 10.1038/s41598-023-27486-7

**Published:** 2023-01-07

**Authors:** Yue Liang, Garvit Bhatt, Lin Tze Tung, HanChen Wang, Joo Eun Kim, Marwah Mousa, Viktoria Plackoska, Katalin Illes, Anna A. Georges, Philippe Gros, Linda Henneman, Ivo J. Huijbers, Bhushan Nagar, Anastasia Nijnik

**Affiliations:** 1 Department of Physiology, McGill University, 368 Bellini Life Sciences Complex, 3649 Promenade Sir William Osler, Montreal, QC H3G 0B1 Canada; 2 McGill University Research Centre on Complex Traits, McGill University, Montreal, QC Canada; 3 Department of Pharmacology, McGill University, Montreal, QC Canada; 4 Department of Biochemistry, McGill University, Montreal, QC Canada; 5 Centre de Recherche en Biologie Structurale (CRBS), McGill University, Montreal, QC Canada; 6 Mouse Clinic for Cancer and Aging, Netherlands Cancer Institute, Antoni Van Leeuwenhoek Ziekenhuis, Amsterdam, The Netherlands

**Keywords:** Haematopoietic stem cells, Haematopoiesis, Leukopoiesis, Lymphopoiesis

## Abstract

Myb-like SWIRM and MPN domains 1 (MYSM1) is a chromatin binding protein with deubiquitinase (DUB) catalytic activity. Rare MYSM1 mutations in human patients result in an inherited bone marrow failure syndrome, highlighting the biomedical significance of MYSM1 in the hematopoietic system. We and others characterized *Mysm1*-knockout mice as a model of this disorder and established that MYSM1 regulates hematopoietic function and leukocyte development in such models through different mechanisms. It is, however, unknown whether the DUB catalytic activity of MYSM1 is universally required for its many functions and for the maintenance of hematopoiesis in vivo. To test this, here we generated a new mouse strain carrying a *Mysm1*^D660N^ point mutation (*Mysm1*^DN^) and demonstrated that the mutation renders MYSM1 protein catalytically inactive. We characterized *Mysm1*^DN/DN^ and *Mysm1*^fl/DN^ Cre^ERT2^ mice, against appropriate controls, for constitutive and inducible loss of MYSM1 catalytic function. We report a profound similarity in the developmental, hematopoietic, and immune phenotypes resulting from the loss of MYSM1 catalytic function and the full loss of MYSM1 protein. Overall, our work for the first time establishes the critical role of MYSM1 DUB catalytic activity in vivo in hematopoiesis, leukocyte development, and other aspects of mammalian physiology.

## Introduction

MYSM1 is primarily a nuclear chromatin binding protein and comprises the Myb-like SANT domain that can bind DNA, the SWIRM domain that is suggested to mediate interactions with histones and other proteins, and the MPN domain with deubiquitinase catalytic activity^1,2^. The MPN metalloprotease domain is characterized by a JAB1-MPN-MOV34 (JAMM) motif, with the consensus sequence (E-[X_2_]-H-S/T-H-[X_7_]-S-[X_2_]-D), which allows MYSM1 to coordinate Zn^2+^ and hydrolyze ubiquitin-linked isopeptide bonds^1,3,4^.

Homozygous or compound heterozygous *MYSM1* mutations in rare human patients result in an inherited bone marrow failure syndrome (IBMFS), characterized by anemia, leukopenia, and in some cases growth delay and mild developmental abnormalities^1,5–7^. In particular, anemia and B lymphocyte depletion of varying severity have been reported in all such patients, NK cell and neutrophil depletion in the majority of the patients, and T lymphocyte depletion less commonly^1,5–7^. Such patients are treated with blood transfusions and hematopoietic stem cell (HSC) transplantation^1,5–7^. We and others characterized *Mysm1*-knockout mice as a model of this disorder, and demonstrated partial embryonic lethality, growth retardation, and complex hematopoietic phenotypes, including loss of HSC quiescence, apoptosis of hematopoietic progenitors, and a severe depletion of lymphoid, erythroid, and other hematopoietic lineages^1,8,9^. All this highlights the importance of MYSM1 in mammalian development and hematopoiesis, and the biomedical significance of understanding its functions.

Monoubiquitinated histone H2A-K119ub was the first MYSM1 substrate to be discovered, and MYSM1 was shown to promote the expression of androgen receptor target genes in prostate cancer cell lines through H2A-K119ub deubiquitination^2^. Similarly, in mouse hematopoietic stem or progenitor cells (HSPCs) MYSM1 was shown to promote the expression of *Gfi1*, *Flt3*, *Id2*, *Ebf1* and *Pax5* genes, important for the normal progression of hematopoiesis, also via H2A-K119ub deubiquitination of their promoters and other regulatory elements^9–13^. With subsequent in vitro assays, MYSM1 was also shown to cleave K63, M1, K6, K27, but not other polyubiquitin chains^14^. In macrophages it dampened inflammatory responses to microbial compounds by removing K63-polyubiquitin from TRAFs, RIP2, and STING proteins in the signal transduction cascades of Toll-like (TLR), NOD2, and cGAS receptors^14–16^. Overall, the emerging evidence indicates that MYSM1 is a multifunctional protein, with multiple substrates for its DUB catalytic activity.

However, it is important to stress that not all MYSM1 functions are directly linked to its DUB catalytic activity, and it also engages in many functionally significant protein–protein interactions^1,2^. Recently, we conducted the first genome-wide analysis for MYSM1-regulated genes in mouse HSCs and reported that MYSM1 maintains the expression of genes encoding ribosomal proteins^17^, however with MYSM1-loss the levels of H2A-K119ub at the MYSM1-binding sites within these gene promoters remained below the level of detection, suggesting other modes for their transcriptional regulation by MYSM1^17^. We and others also characterized p53 activation as the common feature of MYSM1 deficiency and the driving mechanism for hematopoietic failure and other associated pathologies, as demonstrated by the rescue of *Mysm1*^−/−^ phenotypes in the *Mysm1*^−/−^*p53*^−/−^ double knockout mice^18–22^. While several mechanisms for p53 activation in MYSM1 deficiency were proposed, no clear link to MYSM1 DUB catalytic activity has emerged so far^17,19,20,23^. Overall, it remains unknown whether all biologically significant MYSM1 functions are universally dependent on its DUB catalytic activity and whether MYSM1 catalytic activity is essential for the maintenance of hematopoiesis in vivo.

To address these questions, we generated a mouse strain carrying a *Mysm1*^D660N^ point mutation predicted to render MYSM1 protein catalytically inactive (abbreviated *Mysm1*^DN^). We confirm the loss of MYSM1 catalytic activity, and for the first time compare the developmental, hematopoietic, and immune phenotypes resulting from the loss of MYSM1 catalytic function versus the full loss of MYSM1 protein in mouse models.

## Results

### Loss of catalytic activity in the*** MYSM1***^***D660N***^ mutant protein

**Figure 1:**
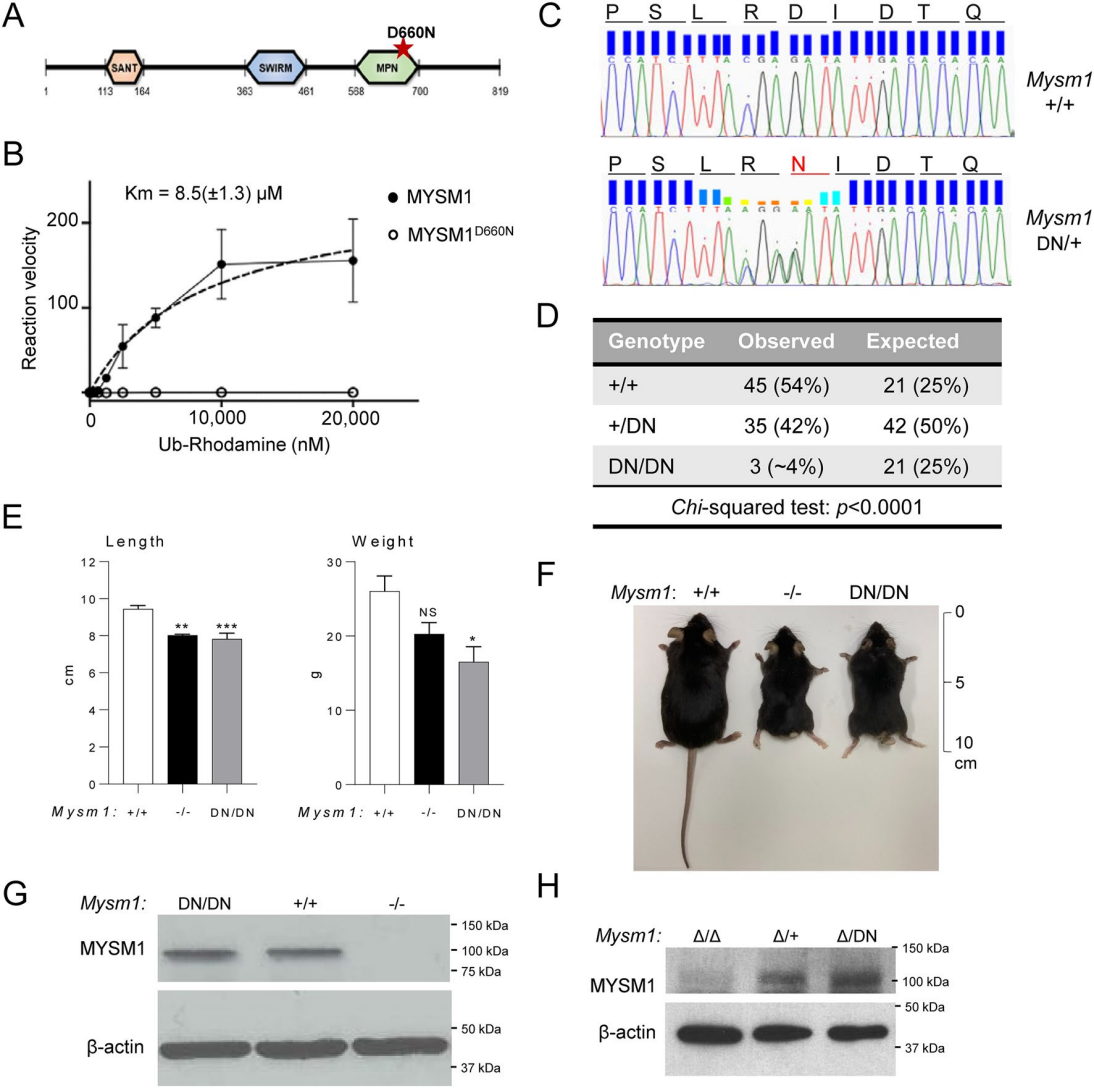
Development and validation of the mouse model expressing a catalytically inactive MYSM1. (**A**) Domain structure of the mouse MYSM1 protein indicating the mutation in the MPN catalytic domain predicted to render the protein catalytically inactive. (**B**) Catalytic activity assay of recombinant mouse MYSM1 against ubiquitin-Rhodamine substrate demonstrates that the D660N mutation results in a full loss of the DUB catalytic activity. (**C**) Sanger sequencing of the *Mysm1* locus in the genomic DNA of wild type control and *Mysm1*^DN/+^ heterozygous mice, indicating the DNA sequences and the corresponding amino acid sequences of the wild type and mutant proteins. (**D**) High embryonic lethality of *Mysm1*^DN/DN^ mice: offspring genotypes obtained from the mating of two *Mysm1*^+/DN^ heterozygous parents show strong deviation from expected Mendelian ratios. (**E**) Length and weight of the age- and sex- matched mice of *Mysm1*^+/+^, *Mysm1*^−/−^ and *Mysm1*^DN/DN^ genotypes; bars represent means ± SEM, statistical analysis with ANOVA comparing each group to the control, *p < 0.05, **p < 0.01, ***p < 0.001, NS—not significant. (**F**) Representative image of the age- and sex- matched mice of *Mysm1*^+/+^, *Mysm1*^−/−^ and *Mysm1*^DN/DN^ genotypes, showing reduced body size and tail dysmorphology. (**G**) Western blot of mouse bone marrow cell lysates showing comparable MYSM1 protein levels in *Mysm1*^+/+^ and *Mysm1*^DN/DN^ samples. (**H**) Western blot of splenocyte lysates from tamoxifen-treated Cre^ERT2^ transgenic mice of *Mysm1*^fl/+^, *Mysm1*^fl/fl^, and *Mysm1*^fl/DN^ genotypes, showing strong depletion of MYSM1 protein in the *Mysm1*^Δ/Δ^ cells, and comparable MYSM1 protein levels between *Mysm1*^Δ/+^ and *Mysm1*^Δ/DN^ samples. β-actin is used as a loading control in (**G**,**H**).

To generate an allele encoding a catalytically inactive MYSM1 in mouse we chose to introduce the *Mysm1*^D660N^ point mutation at the highly conserved aspartic acid 660 residue within the JAMM motif of the MPN catalytic domain (Fig. 1A). This residue is considered essential to the catalytic mechanism and is predicted to interact with both Zn^2+^ and the substrate^3,4^. To confirm that the catalytic activity of MYSM1^D660N^ is indeed impaired, we expressed and purified both wild-type MYSM1 and MYSM^D660N^ proteins from Sf9 insect cells, and performed an in vitro catalytic activity assay using Ubiquitin-Rhodamine 110 as substrate. The proteins were purified from Sf9 insect cells at a similar yield (0.78 mg/L of cells for wild type MYSM1, 0.83 mg/L of cells for MYSM1^D660N^) and eluted in a retention volume slightly lower than 13 mL in size exclusion chromatography, demonstrating that they were not aggregated and likely not misfolded (Fig. S1A). We found that wild-type MYSM1 cleaved the substrate in a dose-dependent manner with a K_m_ of 8.5 μM for the substrate, whereas the activity of MYSM^D660N^ was completely abrogated (Fig. 1B). This confirms that the D660N mutation inactivates the catalytic activity of mouse MYSM1 protein.

### Generation of the*** Mysm1***^***D660N***^ mouse strain

We used CRISPR/Cas9-mediated genome editing in zygotes to introduce the point mutation into exon 16 of *Mysm1*. C57BL/6 zygotes were co-injected with Cas9 protein and a gRNA, along with two homology-dependent recombination (HDR) templates. Since conventional knockout of MYSM1 causes partial embryonic lethality, we used two HDR templates to increase the efficiency of the procedure: one HDR template for the introduction of the D660N mutation and a second template introducing silent mutations to disrupt the gRNA recognition site^24^. The founder harboring both the D660N and silent mutations was backcrossed onto C57BL/6 mice to generate heterozygous *Mysm1*^D660N^ mice lacking the silent mutations on the other *Mysm1* allele. Sanger sequencing of the genomic DNA demonstrated successful introduction of the point mutation that translates into the MYSM1^D660N^ amino acid substitution in the protein (Fig. 1C). The sequencing window covered 371 nucleotides (NCBI GRCm39 *Mysm1*, Gene ID: 320713, range from 94,840,309 to 94,840,679), and included the entire *Mysm1* exon 16 and ≥ 80 nucleotides of the flanking introns at both ends. This demonstrated no other mutations apart from those shown in Fig. 1C and resulting in the D660N substitution in the protein. As the mutations are located > 20 nucleotides away from the 3′ splice site of *Mysm1* exon-16 they are not expected to disrupt splicing^25^; and further analysis of the *Mysm1*^D660N^ allele with the Spliceator online tool (www.lbgi.fr/spliceator/) predicted no changes in splicing. Furthermore, RT-qPCR analysis of mouse bone marrow cells with the primer pairs spanning *Mysm1* exon junctions 15–16 and 16–17 demonstrated no changes in the levels of *Mysm1* transcript successfully spliced across these exon junctions in *Mysm1*^DN/DN^ compared to *Mysm1*^+/+^ control cells (Fig. S1B).

### ***Mysm1***^DN/DN^ mouse model: partial embryonic lethality and developmental phenotypes

*Mysm1*^DN/DN^ mice were born in sub-Mendelian numbers, with only ~ 4% of offspring from an intercross of two heterozygous parents having the *Mysm1*^DN/DN^ genotype, indicating that the loss of MYSM1 catalytic activity causes increased embryonic lethality (Fig. 1D). At adulthood, *Mysm1*^DN/DN^ mice were significantly smaller in length and weight than their littermates (Fig. 1E,F), and had abnormally short tails (Fig. 1F), as previously seen in the *Mysm1*^−/−^ mice^8,9^. Importantly, we demonstrated similar levels of MYSM1 protein in *Mysm1*^DN/DN^ and control *Mysm1*^+/+^ bone marrow cells, and the expected loss of MYSM1 protein expression in *Mysm1*^−/−^ cells (Fig. 1G). Overall, we highlight the similarity in the gross developmental phenotypes of the *Mysm1*^DN/DN^ and *Mysm1*^−/−^ mouse strains, and establish the essential role of the MYSM1 DUB catalytic activity in vivo.

### ***Mysm1***^***fl/DN***^Cre^ERT2^ mouse model for an inducible loss of the MYSM1 catalytic activity

Given the partial embryonic lethality and low availability of the *Mysm1*^DN/DN^ mice, we crossed the mice to the *Mysm1*^fl/fl^Cre^ERT2^ mouse strain that allows highly efficient *Mysm1*^fl^ to *Mysm1*^Δ^ allele conversion with tamoxifen treatment, as demonstrated in our previous studies^17,26^. Here we derived cohorts of Cre^ERT2^-transgenic mice of *Mysm1*^fl/+^, *Mysm1*^fl/fl^, and *Mysm1*^fl/DN^ genotypes, which were born in normal Mendelian numbers, lacked any obvious developmental phenotypes, and bred normally (data not shown). Following tamoxifen treatment, we demonstrated a strong depletion of MYSM1 protein in *Mysm1*^Δ/Δ^ mouse splenocytes, but comparable retention of MYSM1 protein levels in *Mysm1*^Δ/+^ and *Mysm1*^Δ/DN^ samples (Fig. 1H). The Cre^ERT2^ *Mysm1*^fl/DN^ model will test the effects of the loss of MYSM1 DUB catalytic activity on the maintenance of hematopoiesis, leukocyte development, and other aspects of mammalian physiology, independently of its roles in mouse development and the significant developmental phenotypes seen in the *Mysm1*^DN/DN^ mouse strain.

### Severe hematologic dysfunction in*** Mysm1***^DN/DN^ and*** Mysm1***^***fl/DN***^Cre^ERT2^ mice

**Figure 2:**
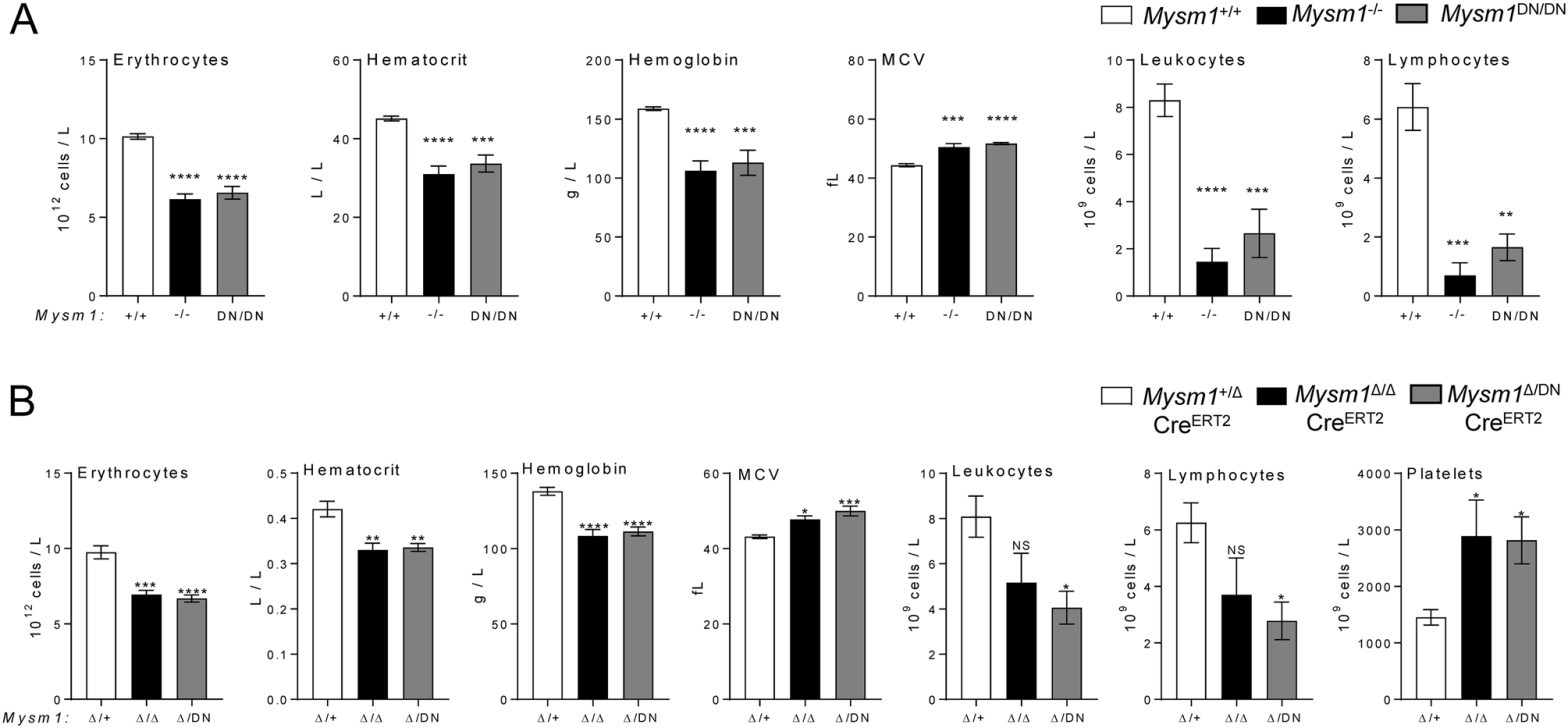
Hematologic dysfunction in the mouse models with the loss of MYSM1 DUB catalytic activity. Hematology analyses were conducted on the blood of (**A**) *Mysm1*^+/+^, *Mysm1*^−/−^, and *Mysm1*^DN/DN^ mice, and (**B**) Cre^ERT2^-transgenic tamoxifen-treated mice of *Mysm1*^fl/+^, *Mysm1*^fl/fl^, and *Mysm1*^fl/DN^ genotypes. Data is from (**A**) 3–8 mice per genotype consolidated from two independent experiments, or (**B**) 5–6 mice per genotype consolidated from two independent experiments. Bars represent means ± SEM; statistical analysis with ANOVA comparing each group to the control, *p < 0.05, **p < 0.01, ***p < 0.001, NS—not significant; MCV—mean corpuscular volume.

Hematology analyses of the blood of *Mysm1*^DN/DN^ and *Mysm1*^−/−^ mice relative to the *Mysm1*^+*/*+^ controls, demonstrated severe hematopoietic dysfunction, characterized by macrocytic anemia, with reduction in blood erythrocyte counts, hematocrit, and hemoglobin concentration, as well as an increased in mean corpuscular volume (MCV, Fig. 2A). Severe depletion of leukocytes and lymphocytes in *Mysm1*^DN/DN^ relative to control *Mysm1*^+*/*+^ mice was also observed (Fig. 2A). Overall, the reported anemia and leukopenia phenotypes of *Mysm1*^DN/DN^ mice are highly consistent with those observed in the *Mysm1*^−/−^ mouse model (Fig. 2A), and also clinically in the patients with *MYSM1* loss-of-function mutations^1,5–7^.

We conducted further hematology analyses on tamoxifen-treated Cre^ERT2^ transgenic mice of *Mysm1*^+/fl^, *Mysm1*^fl/fl^, and *Mysm1*^DN/fl^ genotypes, and observed highly similar hematopoietic phenotypes in the *Mysm1*^Δ/DN^ mice, including macrocytic anemia, leukopenia, and lymphocyte depletion (Fig. 2B). We further observed an increase in platelets in *Mysm1*^DN/Δ^ mice (Fig. 2B), while platelets were not quantified in the *Mysm1*^DN/DN^ model due to increased clotting of the blood samples. Elevated platelet counts were previously reported for the *Mysm1*^−/−^ mouse strain^1,8^, and although the mechanisms remain poorly understood they may be linked to elevated inflammatory response in *Mysm1*^−/−^ mice^14–16^, as thrombocytosis is a common feature of systemic inflammation^27^. Overall, we demonstrate that the loss of MYSM1 DUB catalytic activity in either constitutive or inducible mouse models results in a severe hematologic dysfunction with highly similar phenotypes to the previously characterized *Mysm1*^−/−^ and *Mysm1*^Δ/Δ^ mouse strains.

### Depletion of lymphoid and myeloid immune cells in the*** Mysm1***^***DN/DN***^ mice

**Figure 3:**
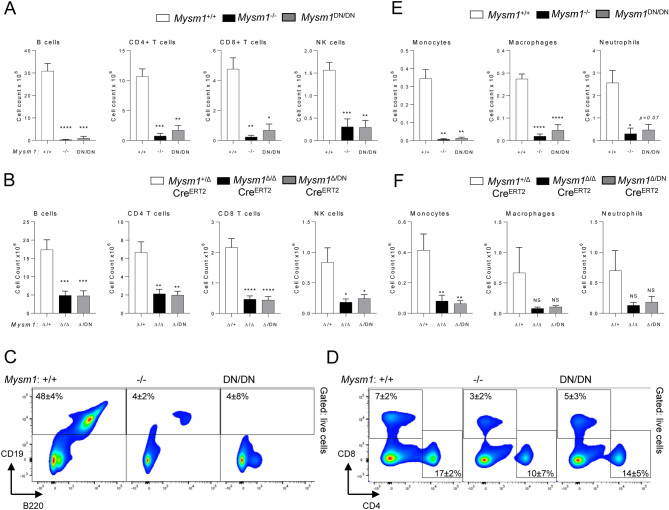
Depletion of splenic lymphoid and myeloid immune cells with the loss of MYSM1 DUB catalytic activity. Flow cytometry analyses were performed on (**A**,**C–E**) *Mysm1*^+/+^, *Mysm1*^−/−^, and *Mysm1*^DN/DN^ mice, and (**B**,**F**) Cre^ERT2^ transgenic mice of *Mysm1*^fl/+^, *Mysm1*^fl/fl^, and *Mysm1*^fl/DN^ genotypes at > 20 weeks after tamoxifen treatment, to quantify (**A**,**B**) B cells (CD19^+^CD3^−^), CD4 T cells (CD3^+^CD4^+^CD8^−^), CD8 T cells (CD3^+^CD4^−^CD8^+^), NK cells (CD3^−^NK1.1^+^), and (**E**,**F**) monocytes (CD45^+^CD3^−^NK1.1^−^CD11b^+^Ly6C^+^Ly6G^−^), macrophages (CD45^+^CD3^−^NK1.1^−^CD11b^+^Ly6G^−^Ly6C^−^F4/80^+^CD64^+^), and neutrophils (CD45^+^CD3^−^NK1.1^−^CD11b^+^Ly6G^+^Ly6C^−^). The data is from (**A,E**) 3–10 mice per genotype consolidated from two independent experiments, or (**B,F**) 8–11 mice per genotype consolidated from three independent experiments. Bars represent means ± SEM; statistical analysis with ANOVA and Dunnett’s post-hoc test, comparing each group to the control, *p < 0.05, **p < 0.01, ***p < 0.001, ****p < 0.0001, NS—not significant. (**C**,**D**) Representative flow cytometry plots of the spleen of *Mysm1*^+/+^, *Mysm1*^−/−^, and *Mysm1*^DN/DN^ mice gated on live cells and showing the depletion of (**C**) CD19^+^ B cells and (**D**) CD4^+^ and CD8^+^ T cells in *Mysm1*^−/−^ and *Mysm1*^DN/DN^ mice; the average frequencies of cells in the gates are presented as mean ± st. dev.

Severe reduction in lymphocyte numbers, including B cells, CD4 T cells, CD8 T cells, and NK cells, was also observed in the spleen of the *Mysm1*^DN/DN^ and *Mysm1*^Δ/DN^ mice, with the overall phenotype being highly similar to that of the *Mysm1*^−/−^ and *Mysm1*^Δ/Δ^ mouse models (Fig. 3A–D). An increase in the proportion of dead cells was also observed, particularly for splenic B cells and NK cells in *Mysm1*^DN/DN^ relative to control *Mysm1*^+/+^ mice (Fig. S2A). Further analyses confirmed the depletion of splenic transitional (T1–3) and follicular B cells in the *Mysm1*^DN/DN^ and *Mysm1*^Δ/DN^ mice, with a somewhat milder depletion of the marginal zone B cell population (Fig. S3A–B). The numbers of myeloid cells, including monocytes, macrophages, and neutrophils, were somewhat more variable across the experimental groups, but also showed a depletion in the *Mysm1*^DN/DN^ and *Mysm1*^Δ/DN^ mice (Fig. 3E,F, Fig. S2B).

### Severe depletion of lymphocyte precursors in the*** Mysm1***^***DN/DN***^ mice

**Figure 4:**
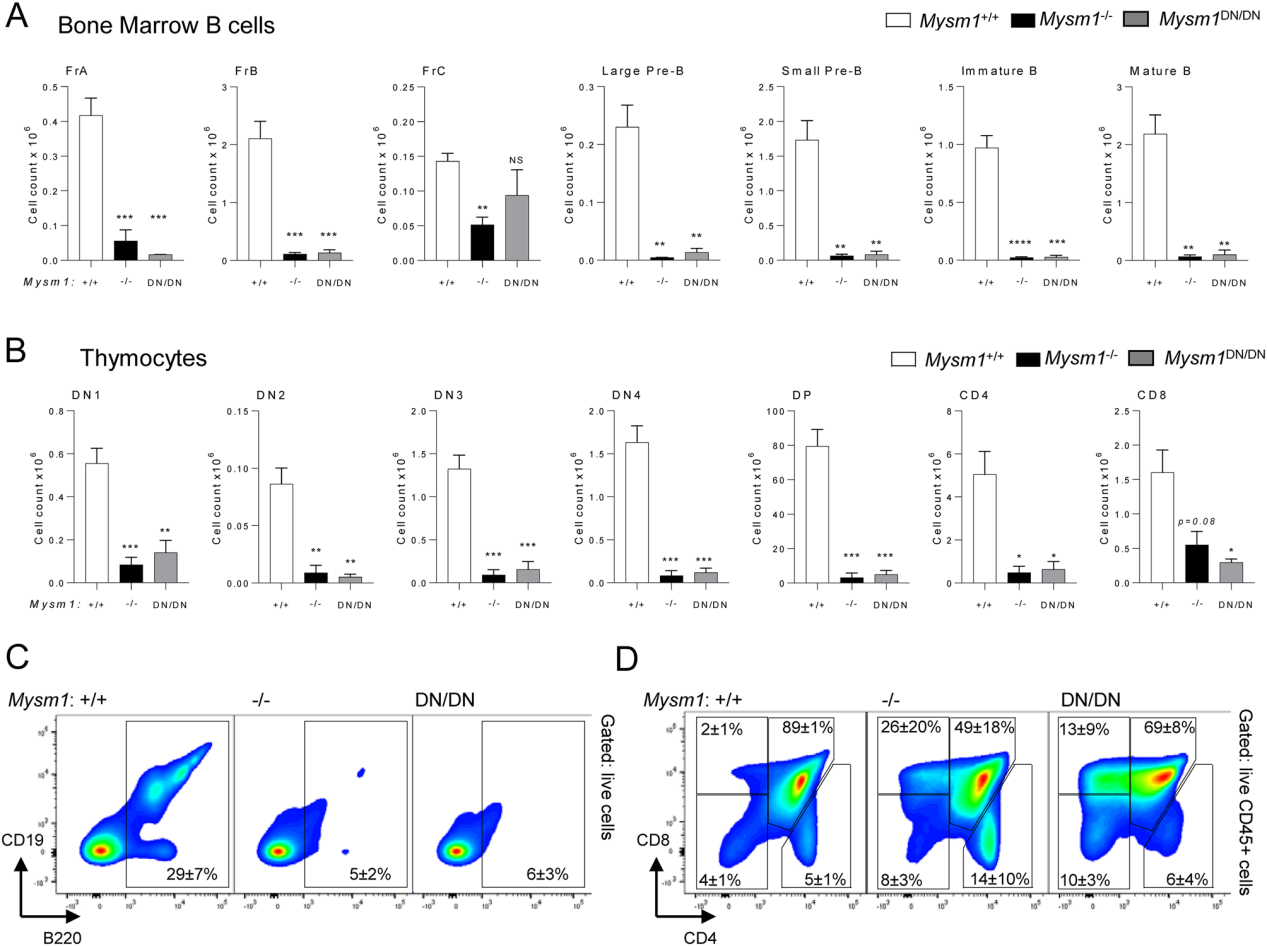
Depletion of B and T lymphocyte precursors in the bone marrow and thymus in mice with the loss of MYSM1 DUB catalytic activity. Flow cytometry analyses were performed on *Mysm1*^+/+^, *Mysm1*^−/−^, and *Mysm1*^DN/DN^ mice, analyzing (**A**) the bone marrow for the following B cell populations: Fraction A (FrA, B220^+^IgM^−^IgD^−^CD43^+^CD24^lo^BP1^lo^), Fraction B (FrB, B220^+^IgM^−^IgD^−^CD43^+^CD24^+^BP1^lo^), Fraction C (FrC, B220^+^IgM^−^IgD^−^CD43^+^CD24^+^BP1^+^), large pre-B cells (B220^+^CD19^+^IgM^−^IgD^−^CD43^−^IL7Rα^hi^FSChi), small pre-B cells (B220^+^CD19^+^IgM^−^IgD^−^CD43^−^IL7Rα^lo^FSC^lo^), immature B cells (B220^+^IgM^+^IgD^−^), mature B cells (B220^+^IgM^+^IgD^+^); and (**B**) the thymus for double negative thymocytes DN1 (CD45^+^CD4^−^CD8^−^CD44^+^CD25^−^), DN2 (CD45^+^CD4^−^CD8^−^CD44^+^CD25^+^), DN3 (CD45^+^CD4^−^CD8^−^CD44^−^CD25^+^), and DN4 (CD45^+^CD4^−^CD8^−^CD44^−^CD25^−^), double positive thymocytes (DP, CD45^+^CD4^+^CD8^+^), and single positive thymocytes (CD45^+^CD4^+^CD8^−^ and CD45^+^CD4^−^CD8^+^). The data is from 3 to 10 mice per genotype consolidated from two independent experiments. Bars represent means ± SEM; statistical analysis with ANOVA and Dunnett’s post-hoc test, comparing each group to the control, *p < 0.05, **p < 0.01, ***p < 0.001; bone marrow cell counts are presented per two tibias and femurs. (**C**) Representative flow cytometry plots of the mouse bone marrow stained for B220 and CD19 B cell markers and (**D**) of the mouse thymus stained for CD4 and CD8; the average frequencies of cells in the gates are presented as mean ± st. dev.

We further analyzed for the B and T cell precursor subsets in the bone marrow and thymus of *Mysm1*^DN/DN^ and *Mysm1*^−/−^ mice, relative to the *Mysm1*^+*/*+^ controls. We observed a strong depletion of most B cell precursor subsets, including pre-pro-B and pro-B cells (Fractions A-B), pre-B cells, and the immature and mature bone marrow B cell populations in the *Mysm1*^DN/DN^ and *Mysm1*^−/−^ mice (Fig. 4A,C). Similarly, we observed a strong depletion of most thymocyte populations in the *Mysm1*^DN/DN^ and *Mysm1*^−/−^ mice (Fig. 4B,D). Overall, this indicates that the loss of MYSM1 DUB catalytic activity or the loss of MYSM1 protein expression both result in a severe defect in B and T lymphocyte development.

### Hematopoietic progenitor depletion and hematopoietic dysfunction in the ***Mysm1***^***DN/DN***^ mice

**Figure 5:**
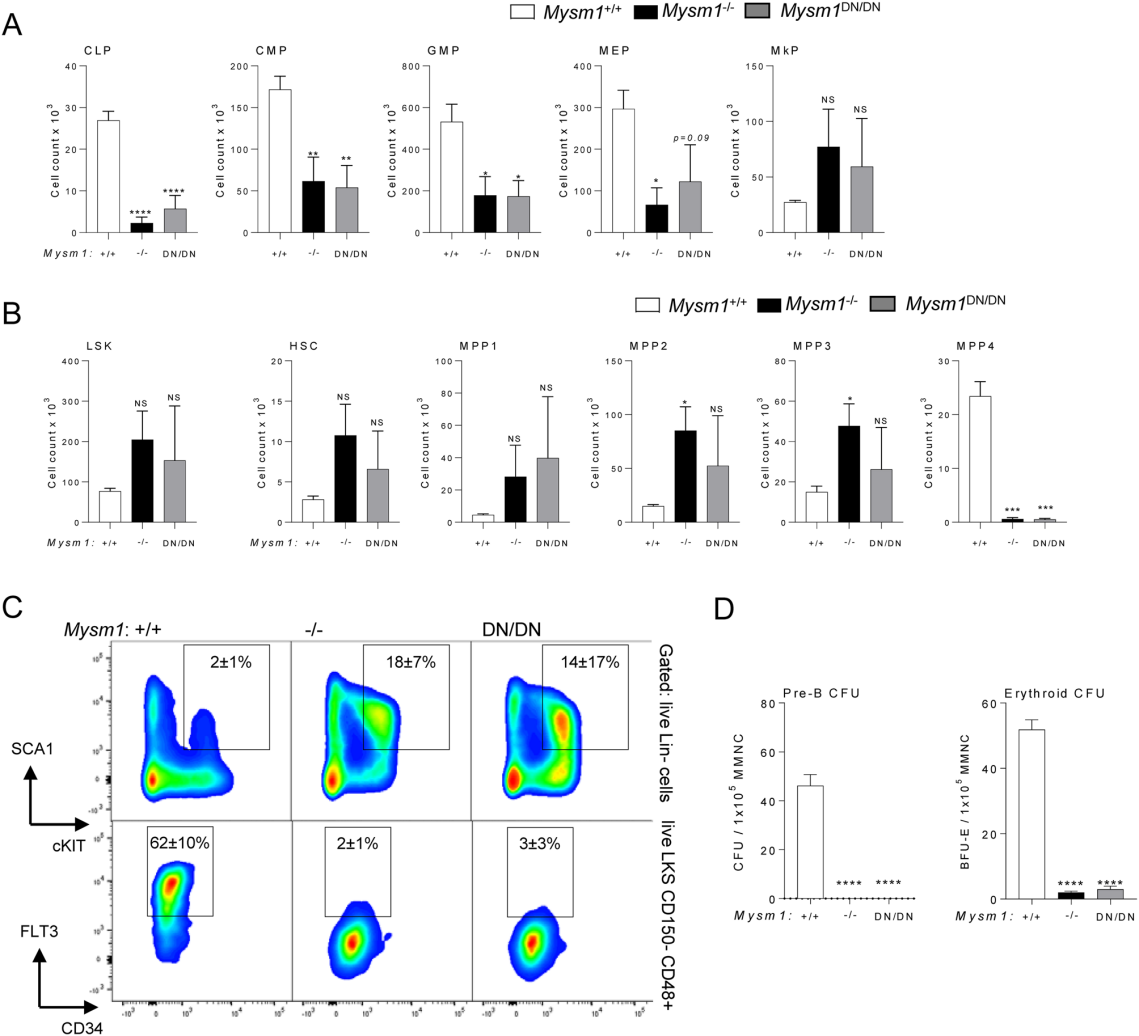
Hematopoietic dysfunction and altered hematopoietic progenitor cell numbers in *Mysm1*^DN/DN^ mice. Flow cytometry analyses were performed on the bone marrow of *Mysm1*^+/+^, *Mysm1*^−/−^, and *Mysm1*^DN/DN^ mice to quantify (**A**) common lymphoid progenitors (CLP, Lin^−^IL7Rα^+^cKit^lo^Sca1^lo^), common myeloid progenitors (CMP, Lin^−^cKit^+^Sca1^−^CD34^+^CD16/32^−^), granulocyte monocyte progenitors (GMP, Lin^−^cKit^+^Sca1^−^CD34^+^CD16/32^+^), megakaryocyte erythroid progenitors (MEP, Lin^−^cKit^+^Sca1^−^CD34^−^CD16/32^−^), and megakaryocyte progenitors (MkP, Lin^−^cKit^+^Sca1^−^CD150^+^CD41^+^); (**B**) hematopoietic stem cells (HSCs) and multipotent progenitors (MPP1-4), gated as LSK (Lin^−^cKit^+^Sca1^+^), followed by CD150^+^CD48^−^CD34^−^Flt3^−^ for HSCs, CD150^+^CD48^−^CD34^+^Flt3^−^ for MPP1, CD150^+^CD48^+^CD34^+^Flt3^−^ for MPP2, CD150^−^CD48^+^CD34^+^Flt3^−^ for MPP3, and CD150^−^CD48^+^CD34^+^Flt3^+^ for MPP4. The data is from 3–10 mice per genotype consolidated from two independent experiments. Bars represent means ± SEM; statistical analysis with ANOVA and Dunnett’s post-hoc test, comparing each group to the control, *p < 0.05, **p < 0.01, ***p < 0.001, ****p < 0.0001, NS—not significant; bone marrow cell counts are presented per two tibias and femurs. (**C**) Representative flow cytometry density plots of the bone marrow of *Mysm1*^+/+^, *Mysm1*^−/−^, and *Mysm1*^DN/DN^ mice, gated on live Lin- cells and showing the LSK cell population (top), or gated on the LSK C150^−^CD48^+^ cells and showing the Flt3^lo^ MPP3 and Flt3^hi^ MPP4 cells; the average frequency of cells in the gates is presented as mean ± st. dev. While the LSK cell numbers are highly variable (top), a strong depletion of the lymphoid-primed MPP4 cells is consistently observed in all the *Mysm1*^−/−^ and *Mysm1*^DN/DN^ mice (bottom). (**D**) Colony forming units (CFU) assays showing depletion of pre-B and erythroid BFU-E progenitors in *Mysm1*^DN/DN^ and *Mysm1*^−/−^ mouse bone marrow; MMNC – marrow mononuclear cells.

To further characterize the dysfunction in hematopoiesis resulting from the in vivo loss of MYSM1 DUB catalytic activity, *Mysm1*^DN/DN^, *Mysm1*^−/−^, and control *Mysm1*^+*/*+^ mice were analyzed for the numbers of hematopoietic progenitor cells across the different lineages, as well as for the multipotent progenitors (MPPs) and hematopoietic stem cells (HSCs). We observed a significant depletion of common lymphoid (CLP), common myeloid (CMP), and granulocyte monocyte (GMP) progenitors in *Mysm1*^DN/DN^ mice (Fig. 5A), while changes in megakaryocyte erythroid (MEP) and megakaryocyte (MkP) progenitors did not reach statistical significance (Fig. 5A). The numbers of HSC and MPP1-3 cells were highly variable between the *Mysm1*^DN/DN^ mice, and showed trends for expansion, which however did not reach statistical significance (Fig. 5B,C), and this likely reflects the competing effects of the loss of HSC quiescence and increased cell apoptosis, as previously reported in the *Mysm1*^−/−^ mouse models^10,19^. Importantly, there was a severe depletion of the lymphoid primed MPP4 cells in both *Mysm1*^DN/DN^ and *Mysm1*^−/−^ relative to control mice (Fig. 5B,C), further supporting the essential role of MYSM1 DUB catalytic activity for lymphopoiesis. Furthermore, an increase in the proportion of dead cells was observed particularly for lymphoid biased MPP4 and CLP cells in *Mysm1*^DN/DN^ relative to control *Mysm1*^+/+^ mice (Fig. S2C-D).

The dysfunction in lymphopoiesis and erythropoiesis in the *Mysm1*^DN/DN^ mice was further demonstrated with colony-forming units (CFU) assays, showing a severe depletion of B-cell lineage and erythroid lineage CFUs in the *Mysm1*^DN/DN^ and *Mysm1*^−/−^ relative to the control mice (Fig. 5D). No analysis of myeloid CFUs was conducted, as in previous studies myeloid CFU numbers in *Mysm1*^−/−^ mice were not significantly impaired^8^.

### Cell intrinsic role of MYSM1 DUB catalytic activity in hematopoiesis

**Figure 6:**
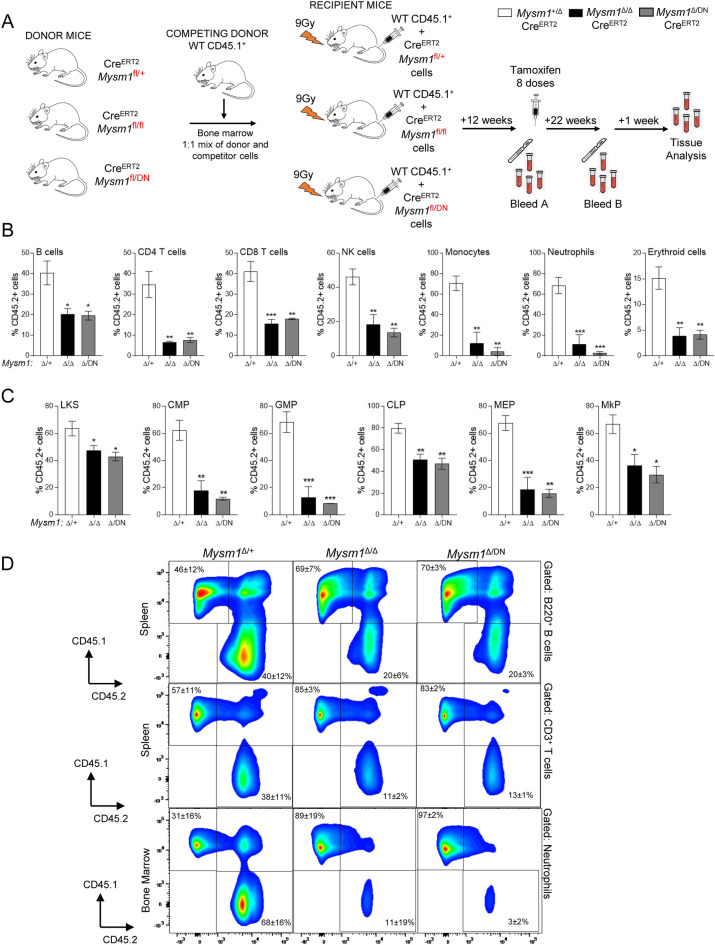
Assessing the cell-intrinsic role of MYSM1 DUB catalytic activity in hematopoiesis and leukocyte development with competitive bone marrow transplantation. (**A**) Schematic representation of the mouse-to-mouse competitive bone marrow transplantation study. Wild type CD45.1^+^ bone marrow cells were mixed in a 1:1 ratio with Cre^ERT2^ transgenic bone marrow cells of *Mysm1*^fl/+^, *Mysm1*^fl/fl^, or *Mysm1*^fl/DN^ genotypes, and the mixes were transplanted into three independent cohorts of lethally irradiated wild type CD45.1^+^ recipient mice. Following full hematopoietic reconstitution, the chimeric mice were administered with tamoxifen to induce the *Mysm1*^fl^ to *Mysm1*^Δ^ allele conversion. Clipart images were used toward the preparation of the Figure (http://clipart-library.com). (**B**,**C**) The relative contribution of *Mysm1*^Δ/DN^*, Mysm1*^Δ/Δ^, and control *Mysm1*^Δ/+^ cells to the different hematopoietic and immune cell populations was evaluated by flow cytometry, quantifying the proportion of CD45.2^+^CD45.1^−^ cells within each cell population. Data is from 3–5 mice per group; bars represent means ± SEM; statistical analysis uses ANOVA and Dunnett’s post-hoc test comparing each group to the *Mysm1*^Δ/+^ control; *p < 0.05, **p < 0.01, ***p < 0.001, or NS—not significant. Data is presented for the following cell populations: (**B**) splenic B cells (CD19^+^CD3^−^), CD4 T cells (CD3^+^CD4^+^CD8^−^), CD8 T cells (CD3^+^CD4^−^CD8^+^), and NK cells (CD3^−^NK1.1^+^); bone marrow monocytes (CD11b^+^Ly6C^hi^Ly6G^lo^), neutrophils (CD11b^+^Ly6C^lo^Ly6G^hi^), and erythroid cells (CD71^+^); (**C**) bone marrow stem and multipotent progenitors (LKS, Lin^−^cKit^+^Sca1^+^), common myeloid progenitors (CMP, Lin^−^cKit^+^Sca1^−^CD34^+^CD16/32^−^), granulocyte monocyte progenitors (GMP, Lin^−^cKit^+^Sca1^−^CD34^+^CD16/32^+^), common lymphoid progenitors (CLP, Lin^−^cKit^lo^Sca1^lo^IL7Ra^+^CD16/32^−^), megakaryocyte erythroid progenitors (MEP, Lin^−^cKit^+^Sca1^−^CD34^−^CD16/32^−^), and megakaryocyte progenitors (MkP, Lin^−^cKit^+^Sca1^−^CD16/32^−^CD150^+^CD41^+^). (**D**) Representative flow cytometry plots showing the analyses of splenic B cells, splenic T cells, and bone marrow neutrophils for CD45.1 versus CD45.2 marker expression; percentage of cells in the gates is shown as mean ± st. dev. of all the mice in each group. Gates for CD45.1^+^ and CD45.2^+^ cells were set independently for each cell population using control non-chimeric WT-B6 (CD45.2) and WT-SJL (CD45.1) mice, as shown in Fig. S4E.

To directly test the cell-intrinsic requirement for the MYSM1 DUB catalytic activity in hematopoiesis, competitive bone marrow chimeras were set up. CD45.1^+^ wild type bone marrow was mixed in a 1:1 ratio with the bone marrow of CD45.2^+^ Cre^ERT2^ mice of *Mysm1*^+/fl^, *Mysm1*^fl/fl^, or *Mysm1*^DN/fl^ genotypes, and transplanted into three independent groups of lethally irradiated recipients (Fig. 6A). The recipient mice were bled at 12-weeks to confirm the normal reconstitution with donor bone marrow across the genotypes (data not shown), and subsequently all the mice were administered with tamoxifen to induce the *Mysm1*^fl^ to *Mysm1*^Δ^ allele conversion. The mice were analyzed for the relative contributions of the CD45.2^+^ bone marrow to the different hematopoietic lineage, across the three *Mysm1* genotypes.

We observed a significant reduction in the contribution of the *Mysm1*^DN/Δ^ donor hematopoiesis to the B cell, CD4 T cell, CD8 T cell, and NK cell populations in the mouse spleen (Fig. 6B), to monocyte and neutrophil populations in both spleen and bone marrow (Fig. 6B and not shown), and to all the leukocyte populations in the mouse blood (Fig. S4A). Similar defects in the reconstitution were observed for the *Mysm1*^DN/Δ^ hematopoietic progenitor cells, including the lineage committed progenitors (CMPs, GMPs, CLPs, MEPs, and MkPs, Fig. 6C), all the developing B cell subsets (Fractions A-C, pre-B, and immature B cells, Fig. S4B), and the majority of T cell precursor subsets within the thymus (Fig. S4C). Among the multipotent HSC and MPP hematopoietic cells there was no defect in *Mysm1*^DN/Δ^ reconstitution of the early HSC and MPP1-2 cells, likely reflecting the balancing effects of the loss of quiescence and increase in apoptosis among these cells, as in the *Mysm1*^−/−^ mouse models^10,19^, however impaired reconstitution was seen for the latter myeloid-biased MPP3 and lymphoid-biased MPP4 subsets (Fig. S4D). Importantly, throughout the datasets presented above the *Mysm1*^DN/Δ^ phenotypes aligned very well with the *Mysm1*^Δ/Δ^ group, both showing strong impairment of hematopoietic function relative to the *Mysm1*^+/Δ^ control (Fig. 6, S4). Overall, this demonstrates the essential and cell-intrinsic role of the MYSM1 DUB catalytic activity in the regulation of hematopoiesis, and suggests lack of significant MYSM1 mechanisms of action that are independent of its catalytic function.

## Discussion

In this study, we for the first time establish and characterize a mouse strain expressing a catalytically inactive MYSM1^D660N^ protein. We demonstrate a profound similarity in the developmental, hematopoietic, and immune phenotypes of *Mysm1*^Δ^ and *Mysm1*^DN^ mice, indicating the critical role of MYSM1 DUB catalytic activity in hematopoiesis and other aspects of mammalian physiology. While the depletion of hematopoietic cells and HSPC failure in functional assays were highly consistent between the *Mysm1*^−/−^ and *Mysm1*^DN/DN^ strains throughout this study, the *Mysm1*^DN/DN^ strain showed high embryonic lethality, with only ~ 4% of *Mysm1*^DN/DN^ offspring from an intercross of heterozygous parents, as compared to 10% for the *Mysm1*^−/−^ strain reported in previous studies^8^. Although these figures are not directly comparable between studies, it is interesting to note that increased embryonic lethality has been reported in other strains expressing catalytically inactive proteins relative to the corresponding knockout strains^28,29^.

While the current study provides an in-depth analysis of the role of MYSM1 DUB catalytic activity in hematopoiesis and leukocyte development, the International Mouse Phenotyping consortium^30,31^ and previously published studies report complex phenotypes in many other physiological systems in *Mysm1*^−/−^ mice, including alterations in skeletal, skin, and adipose physiology^21,22,32,33^. In future work, a broader comparison of *Mysm1*^−/−^ and *Mysm1*^DN/DN^ mouse strains will allow us to further explore the role of MYSM1 catalytic activity in these other physiological systems. Recent studies also established MYSM1 as a negative regulator of inflammatory responses to microbial stimuli in macrophages^14–16^. In these studies MYSM1 was shown to deubiquitinate TRAFs, RIP2, and STING proteins in the signal transduction cascades of innate immunity^14–16^. The *Mysm1*^DN/DN^ mouse strain developed in our current work may be used to further validate the role of MYSM1 catalytic activity in the regulation of innate immune and inflammatory responses in vivo.

Although no molecular analyses for the mechanisms of hematopoietic failure were conducted in the *Mysm1*^DN/DN^ and *Mysm1*^DN/Δ^ mouse models in the current study, the very high concordance of their phenotypes to those of the *Mysm1*^−/−^ and *Mysm1*^Δ/Δ^ mouse strains suggests that similar molecular mechanisms are at play. Previously in the *Mysm1*^−/−^ mice the hematopoietic failure was shown to be driven by the activation of p53 and the induction of its pro-apoptotic transcriptional programs^18–20^. Consistently, our current data showed some increase in cell death in *Mysm1*^DN/DN^ relative to control *Mysm1*^+/+^ hematopoietic and immune cells, supporting that similar mechanisms may also mediate the hematopoietic failure in *Mysm1*^DN/DN^ mice.

With the validation of the role of MYSM1 DUB catalytic activity in hematopoiesis, it will be important to identify the full range of protein targets and substrates of MYSM1, for example through proteomics approaches^34–36^. While histone H2A-K119ub is a highly abundant and well characterized MYSM1 substrate^2,37^, MYSM1 can also cleave K63, M1, K6, and K27 polyubiquitin in vitro^14^ and regulates K63-polyubiquitination of TRAFs, RIP2, and STING proteins in macrophages^14–16^. Given the diverse and complex roles of ubiquitination in regulating chromatin accessibility, gene expression, genomic stability, signal transduction, protein localization and many other cellular processes^37–39^, such studies may lead to the discovery of further novel MYSM1 substrates beyond histone H2A-K119ub and advance the understanding of its functions and mechanisms of action.

We recently established that the loss of MYSM1 in mouse models of cMYC-driven B cell lymphoma can protect against disease onset and progression^40^. At the cellular and molecular levels, the protective effects were attributed to the role of MYSM1 in the cMYC-dependent induction of the genes encoding ribosomal proteins in the tumor cells (*Rps*/*Rpl* genes), with MYSM1-loss resulting in reduced *Rps*/*Rpl* transcript levels, reduced cellular protein synthesis rates, and the activation of p53 tumour suppressor^40^. Overall, these studies may suggest MYSM1 as a drug-target for cMYC driven hematologic malignancies; and the *Mysm1*^DN^ mouse strain described in our current work will allow to test whether the loss of MYSM1 DUB catalytic function can offer similar therapeutic benefits. This can serve as a proof-of-concept for the development of pharmacological MYSM1 inhibitors and for the assessment of their activities in experimental models of cMYC-driven hematologic malignancies.

In summary, our study establishes the primary and indispensable function of MYSM1 as a DUB in vivo in the normal progression of mammalian development, hematopoiesis, and immune cell production. This work also provides a mouse model for further analyses of the roles of MYSM1 DUB catalytic functions in vivo in many other aspects of mammalian physiology.

## Materials and methods

### Generation of ***Mysm1***^***D660N***^ mice

*Mysm1*^D660N^ mice were generated on a C57BL/6JRj background using pronuclear microinjection in mouse zygotes. Zygotes isolated from C57BL/6JRj mice were co-injected with Cas9 protein (200 ng/µl) and *Mysm1* exon 16-targeting guide RNA (5′-GTGTCAATATCTCGTAAAGA-3′; 25 ng/µl) from px330 plasmid, along with oligonucleotide repair templates (20 ng/µl) for the introduction of the D660N mutation (see below). Founder mice were screened for the desired point mutation by PCR amplification and Sanger sequencing using the following primers: 5′-GGCATTATAGTGCACTCTGGAA-3′ and 5′-TATACTCAACTGCTGACCTTCCA-3′. *Mysm1*^D660N^ founders were backcrossed once onto C57BL/6JRj and maintained for the desired experimental genotype.

ssDNA D660N HDR repair template:

GGCTACAGTGTCATTGGGTGGTACCATTCTCATCCTGCATTTGATCCTAATCCATCTTTAAGGAATATTGACACACAAGCCAAATACCAGGTGTGTTGTTACATACCTACATTTTGTAAATTATTA

ssDNA silent mutation HDR repair template:

GGCTACAGTGTCATTGGGTGGTACCATTCTCATCCTGCATTTGATCCTAATCCATCTTTAAGGGACATTGACACACAAGCCAAATACCAGGTGTGTTGTTACATACCTACATTTTGTAAATTATTA

### Other mouse lines and genotyping

Mouse lines *Mysm1*^−/−^ and *Mysm1*^fl/fl^ carry the loss-of-function and the conditional alleles of *Mysm1* gene, respectively, and were previously described^8,26,41^. *Mysm1*^fl/fl^ *Cre*^ERT2^ mice were derived for tamoxifen-inducible *Mysm1* deletion by crossing *Mysm1*^fl/fl^ and Gt(ROSA)26Sor^tm1(cre/ERT2)^ strains, as previously described^26^. All lines were on the C57BL/6 genetic background. The mice were maintained under specific pathogen-free conditions and sex-matched across experimental groups. All experiments were in accordance with the guidelines of the Canadian Council on Animal Care and protocol AUP-2011–6029 approved by the McGill Animal Care Committee.

Mouse genotyping for the *Mysm1*^D660N^ allele was performed with a custom designed TaqMan SNP Genotyping assay and TaqMan Genotyping Master Mix on a StepOnePlus instrument (all reagents from ThermoFisher Scientific). Other genotyping was performed by conventional genomic PCR with DreamTaq DNA Polymerase (ThermoFisher Scientific) and primers from Integrated DNA Technologies.

### Tamoxifen mouse treatment

For tamoxifen-induced *Mysm1*-gene deletion, mice of *Mysm1*^fl/fl^ *Cre*^ERT2^ and control genotypes were injected intraperitoneally with tamoxifen (Sigma-Aldrich, T5648) in sterilized corn oil at 0.12 mg per gram body weight per injection, with 8 doses administered in total over 16 days, as in our previous work^17,26,42^. Successful deletion of *Mysm1* exon 3 was validated by PCR analyses of the genomic DNA from lymphoid organs of the mice, as described previously^26,42^.

### Mouse bone marrow transplantation

For competitive bone marrow transplantations, recipient wild type B6.SJL-PtprcaPepcb/Boy (JAX002014, congenic for CD45.1) mice were irradiated with 2 doses of 4.5 Gy, delivered 3 h apart, in an RS2000 irradiator (Rad Source). Wild-type CD45.1-marked bone marrow cells were mixed in a 1:1 ratio with bone marrow cells from mice of *Mysm1*^fl/+^*Cre*^ERT2^, *Mysm1*^fl/fl^*Cre*^ERT2^, or *Mysm1*^fl/DN^*Cre*^ERT2^ genotypes, and the mixes transplanted into three independent cohorts of recipient mice via intravenous injection. The mice were kept on neomycin in drinking water (2 g/l, BioShop) for 3 week. Successful reconstitution of the hematopoietic system by donor cells was confirmed with a bleed and flow cytometry analysis at 12 weeks, and was followed with tamoxifen treatment to induce *Mysm1*^fl^ to *Mysm1*^Δ^ allele conversion and further studies to compare hematopoietic function across the *Mysm1* genotypes.

### Flow cytometry

Cell suspensions of mouse tissues were prepared in RPMI-1640 (Wisent) with 2% (v/v) fetal calf serum (FCS), 100 μg/ml streptomycin and 100U/ml penicillin (Wisent). The cells were stained for surface-markers in PBS with 2% FCS for 20 min on ice, using antibodies listed in Supplemental Table S1. Viability Dye eFluor^®^ 506 (ThermoFisher Scientific) was used to discriminate dead cells. Compensation was performed with BD™ CompBeads (BD Biosciences). The data were acquired on BD Fortessa and analyzed with FlowJo (Tree Star) software.

### Western blotting

Western blotting was performed as previously described^19^, with cells lysed in RIPA buffer supplemented with protease and phosphatase inhibitors (ThermoFisher Scientific). Protein concentration was measured using the BCA assay (ThermoFisher Scientific) on an EnSpire 2300 multilabel reader (PerkinElmer). Protein lysate samples were boiled in Laemmli buffer and 1.25% β-mercaptoethanol (Sigma-Aldrich) before loading onto gels, alongside Precision Plus Kaleidoscope standards (Bio-Rad). Upon gel-to-membrane transfer, nitrocellulose membranes (GE Healthcare) were blocked with 5% milk in TBS-T and probed with antibodies against MYSM1 (EPR18657, Abcam) and β-Actin (D6A8, Cell Signaling Technology) at 4 °C overnight, followed by secondary antibodies Abcam—goat anti-rabbit IgG H&L (HRP)—ab6721 horseradish peroxidase (HRP)-conjugated or Rockland—Rabbit TrueBlot: anti-rabbit IgG HRP—18–8816-31 at room temperature for 1 h, with TBS-T washes after each incubation. Protein bands were detected using Western Lightning Plus-ECL (PerkinElmer) and HyBlot CL autoradiography films (Harvard Apparatus Canada).

### RNA Isolation and RT-qPCR

RNA was extracted using the MagMAX™ Total RNA Isolation Kit (Invitrogen, Thermo Fisher Scientific) according to manufacturer’s protocol, and quantified with NanoDrop (ThermoFisher Scientific). cDNA was prepared using the Moloney murine leukemia virus (MMLV) reverse-transcription kit and quantitative PCRs run on a StepOnePlus instrument with PowerSYBR master mix (all from ThermoFisher Scientific). Primers were purchased from Integrated DNA Technologies, and the sequences are provided in Supplemental Table S2.

### In vitro fluorescence catalytic activity assay

Genes for wild-type and mutated mouse MYSM1 were sub-cloned into pFastBac vectors, which contain an N-terminal hexahistidine tag. The D660N mutation was introduced using standard site-directed mutagenesis protocols. MYSM1 proteins were expressed in Sf9 cells, infected with recombinant baculovirus and grown at 27$^\circ$C for ~ 66 h. Proteins were purified by sonicating the cells, followed by Ni–NTA affinity, Mono-Q and size exclusion chromatography (Superdex 200i). The final yield was ~ 0.8 mg/ L of cells.

Enzymatic assays were conducted in assay buffer comprised of 20 mM Tris pH 8.1, 100 mM NaCl, 0.01% BSA, 1 mM DTT at room temperature in 96-well low binding black plates. 12.5 μL of either MYSM1 or MYSM1^D660N^ at 300 nM was added to the 96-well plate followed by the addition of 12.5μL of serially diluted substrate (0.02—40 μM) and immediately placed into a fluorescence spectrophotometer with excitation/emission wavelengths set to 485/535 nm, respectively.

### Statistics

Statistical analyses used Prism 7.01 (GraphPad Inc.), with Student’s two-tailed *t*-test for two datasets and ANOVA for multiple comparisons; *p* < 0.05 was considered significant.

### Ethical approval

Mouse experiments were in accordance with the guidelines of the Canadian Council on Animal Care and the protocol AUP-2011-6029 approved by the McGill Animal Care Committee.

### ARRIVE guidelines statement

The study is reported in accordance with ARRIVE guidelines. Experimental unit corresponds to a single animal. Mice used in the study included both males and females and were always sex-matched between the groups. Mice were also age-matched between the groups and were at least 8 weeks of age, however in the studies involving lengthy tamoxifen treatments and bone marrow transplantations they were significantly older at the endpoint of the study. Whenever possible mice of test and control groups were bred as litter-mates and maintained in shared cages. Mice were allocated to experimental groups based on genotype and with no randomization; staff carrying out the experiments was not blinded to group allocations; no a priori sample size calculations were performed.

## Supplementary Information

Supplementary Information 1.

Supplementary Information 2.

Supplementary Information 3.

## Data Availability

The relevant datasets used and analyzed during the current study are available from the corresponding author on reasonable request.
