# Supplementary material for: Deubiquitinase catalytic activity of MYSM1 is essential in vivo for hematopoiesis and immune cell development
Source: Sci Rep. 2023 Jan 7;13:338. doi: 10.1038/s41598-023-27486-7 (PMC9825392; doi:10.1038/s41598-023-27486-7)
Supplement: Supplementary file 1 — Supplementary Information 1. [file 41598_2023_27486_MOESM1_ESM.pdf]

SUPPLEMENTAL MATERIALS

Deubiquitinase catalytic activity of MYSM1 is essential in vivo for hematopoiesis and immune cell development

Yue Liang [1,2], Garvit Bhatt [3,4], Lin Tze Tung [1,2], HanChen Wang [1,2], Joo Eun (June) Kim [1,2], Marwah Mousa [1,2], Viktoria Plackoska [1,2], Katalin Illes [4,5], Anna A. Georges [2,4], Philippe Gros [2,4], Linda Henneman [6], Ivo J. Huijbers [6], Bhushan Nagar [4,5], Anastasia Nijnik [1,2,*]

[1] Department of Physiology, McGill University, Montreal, QC, Canada
[2] McGill University Research Centre on Complex Traits, McGill University, QC, Canada
[3] Department of Pharmacology, McGill University, Montreal, QC, Canada
[4] Department of Biochemistry, McGill University, Montreal, QC, Canada
[5] Centre de Recherche en Biologie Structurale (CRBS), McGill University, Montreal, QC, Canada
[6] Mouse Clinic for Cancer and Aging, Netherlands Cancer Institute, Antoni van Leeuwenhoek Ziekenhuis, Amsterdam, Netherlands

***Corresponding author:** Anastasia Nijnik, 368 Bellini Life Sciences Complex, 3649 Promenade Sir William Osler, McGill University, H3G 0B1 Montreal, QC, Canada. Tel: 1-514-398-5567, Fax: 1-514-398-2603, Email: anastasia.nijnik@mcgill.ca.

SUPPLEMENTAL FIGURES

Figure S1. Supplemental analyses of MYSM1^{D660N} protein and transcript. (A) Size exclusion chromatography of murine wild type MYSM1 (green) and MYSM1^{D660N} (purple) proteins, recombinantly expressed in Sf9 insect cells, with associated SDS-PAGE. The Superdex 200 increase analytical column (GE Healthcare) was eluted at a flow rate of 0.75 mL/min with 20 mM Tris (pH 8.1), 150 mM NaCl, 1 mM DTT. Fractions containing the cleanest samples were pooled and concentrated to ~10 mg/mL. The yields of the wild type and mutant proteins were 0.78 mg/L and 0.83 mg/L of insect cell culture, respectively. **(B)** RT-qPCR analysis of the *Mysm1* transcript in mouse bone marrow cells using new primer pairs spanning the *Mysm1* exon junctions 15-16 and 16-17, demonstrating no change in the levels of *Mysm1* transcript spliced across these exon junctions in *Mysm1*$^{DN/DN}$ relative to *Mysm1*$^{+/+}$ control cells. Each dot represents a different mouse; transcript levels are normalized to *Hprt* housekeeping control transcript and then to the mean *Mysm1* transcript levels in the *Mysm1*$^{+/+}$ control mice.

Figure S1

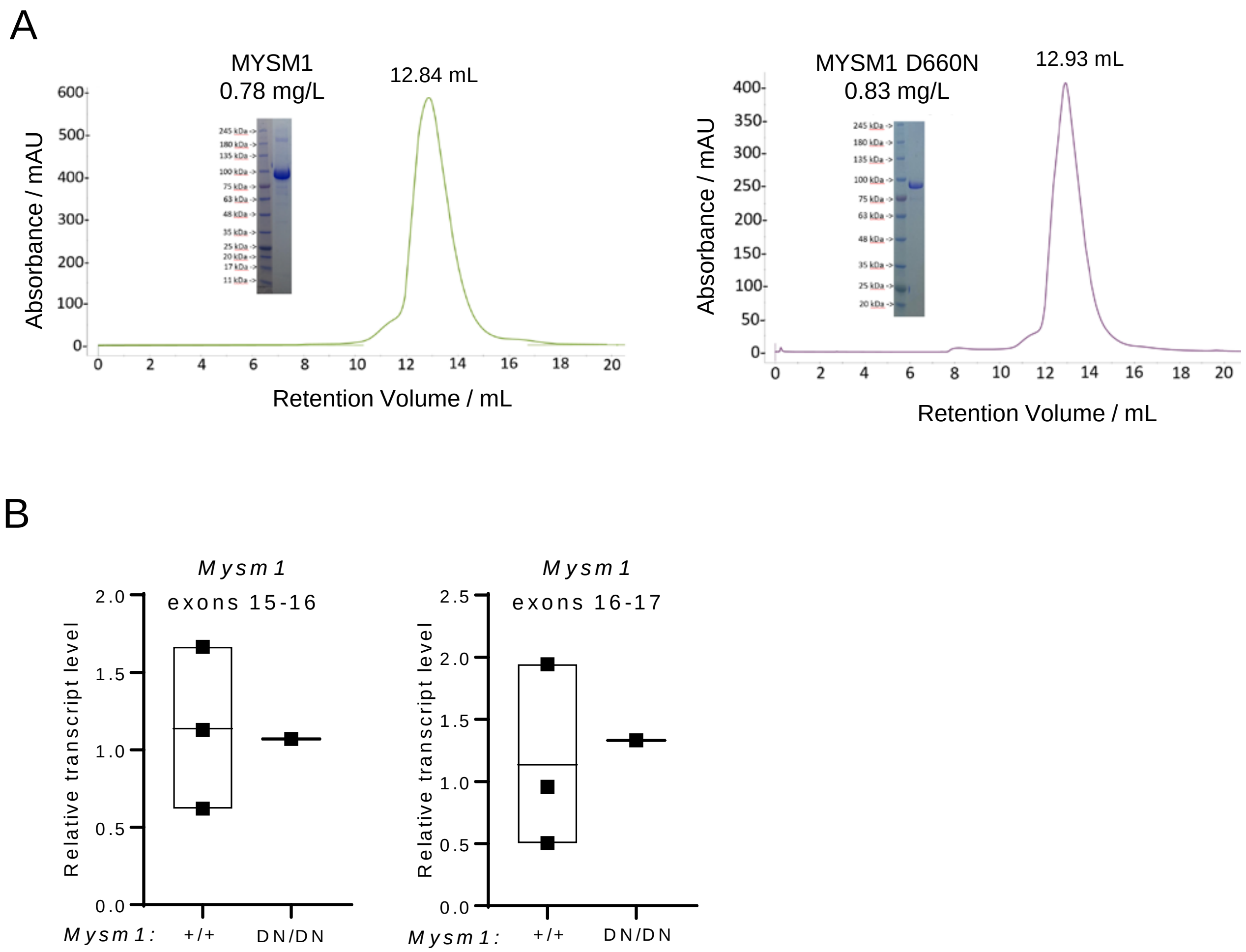

A
MYSM1
0.78 mg/L
12.84 mL
600
500
400
300
200
100
0
Absorbance / mAU
0
2
4
6
8
10
12
14
16
18
20
Retention Volume / mL
MYSM1 D660N
0.83 mg/L
12.93 mL
400
350
300
250
200
150
100
50
0
Absorbance / mAU
0
2
4
6
8
10
12
14
16
18
20
Retention Volume / mL
B
Mysm1
exons 15-16
2.0
1.5
1.0
0.5
0.0
Relative transcript level
Mysm1:
+/+
DN/DN
Mysm1
exons 16-17
2.5
2.0
1.5
1.0
0.5
0.0
Relative transcript level
Mysm1:
+/+
DN/DN

Figure S2. Supplemental analyses of the proportion of dead cells among hematopoietic and immune cells in *Mysm1*$^{DN/DN}$ relative to control *Mysm1*$^{+/+}$ mice, based on eFluor506 viability dye staining and flow cytometry. (A-B) Splenic immune cells were gated as follows: B cells (CD19^{+}CD3^{-}), CD4 T cells (CD3^{+}CD4^{+}CD8^{-}), CD8 T cells (CD3^{+}CD4^{-}CD8^{+}), NK cells (CD3^{-}NK1.1^{+}), monocytes (CD45^{+}CD3^{-}NK1.1^{-}CD11b^{+}Ly6C^{+}Ly6G^{-}), macrophages (CD45^{+}CD3^{-}NK1.1^{-}CD11b^{+} Ly6G^{-}Ly6C^{-}F4/80^{+}CD64^{+}), and neutrophils (CD45^{+}CD3^{-}NK1.1^{-}CD11b^{+}Ly6G^{+}Ly6C^{-}). **(C)** Bone marrow hematopoietic stem cells and multipotent progenitor cells were gated as LSK (Lin^{-}cKit^{+}Sca1^{+}), followed by CD150^{+}CD48^{-}CD34^{-}Flt3^{-} for HSCs, CD150^{+}CD48^{-}CD34^{+}Flt3^{-} for MPP1, CD150^{+}CD48^{+}CD34^{+}Flt3^{-} for MPP2, CD150^{-}CD48^{+}CD34^{+}Flt3^{-} for MPP3, and CD150^{-}CD48^{+}CD34^{+}Flt3^{+} for MPP4. **(D)** Bone marrow lineage committed progenitors were gated as follows: common lymphoid progenitors (CLP, Lin^{-}IL7Rα^{+}cKit^{lo}Sca1^{lo}), common myeloid progenitors (CMP, Lin^{-}cKit^{+}Sca1^{-}CD34^{+}CD16/32^{-}), granulocyte monocyte progenitors (GMP, Lin^{-}cKit^{+}Sca1^{-}CD34^{+}CD16/32^{+}), megakaryocyte erythroid progenitors (MEP, Lin^{-}cKit^{+}Sca1^{-}CD34^{-}CD16/32^{-}), and megakaryocyte progenitors (MkP, Lin^{-}cKit^{+}Sca1^{-}CD150^{+}CD41^{+}). Data is from 3 *Mysm1*$^{DN/DN}$ and 10 *Mysm1*$^{+/+}$ control mice consolidated from two independent experiments. Bars represent means ± SEM; statistical analyses with unpaired Student's *t*-test, * $p<0.05$, ** $p<0.01$, *** $p<0.001$, NS - not significant.

Figure S2

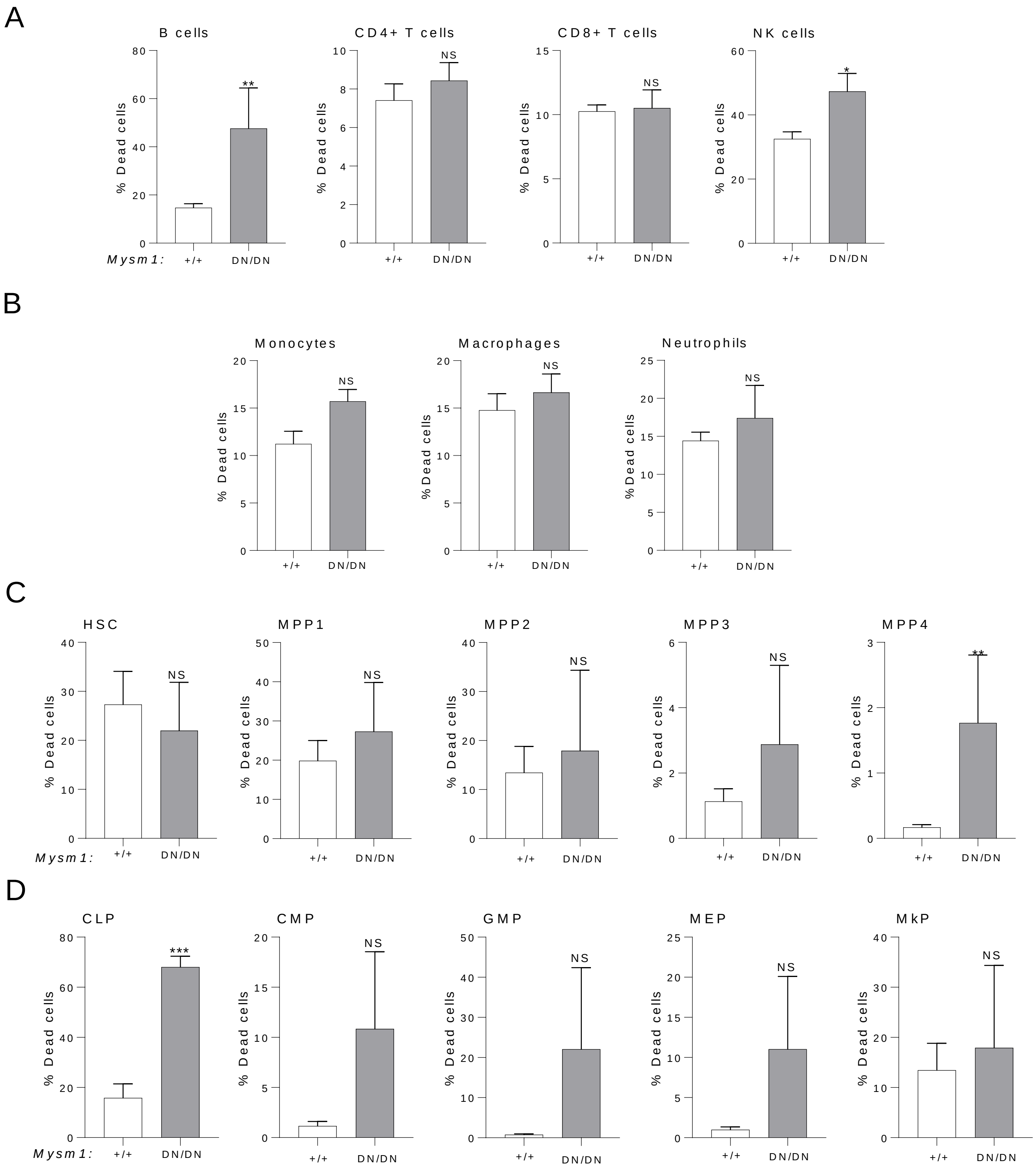
A
B cells
CD4+ T cells
CD8+ T cells
NK cells
% Dead cells
**
NS
NS
*
Mysm1:
+/+
DN/DN
B
Monocytes
Macrophages
Neutrophils
NS
C
HSC
MPP1
MPP2
MPP3
MPP4
D
CLP
CMP
GMP
MEP
MkP
***

Figure S3. Depletion of splenic transitional, follicular, and marginal zone B cells with the loss of MYSM1 DUB catalytic activity. Flow cytometry analyses were performed on **(A)** *Mysm1*$^{+/+}$, *Mysm1*$^{-/-}$, and *Mysm1*$^{DN/DN}$ mice, and on **(B)** Cre^{ERT2} transgenic mice of *Mysm1*$^{fl/+}$, *Mysm1*$^{fl/fl}$, and *Mysm1*$^{fl/DN}$ genotypes at >20 weeks after tamoxifen injections, to quantify the following B cell populations: transitional 1 (T1, CD19^{+}B220^{+}CD93^{+}IgM^{+}CD23^{-}), transitional 2 (T2, CD19^{+}B220^{+}CD93^{+}IgM^{+}CD23^{+}), transitional 3 (T3, CD19^{+}B220^{+}CD93^{+}IgM^{lo}CD23^{+}), follicular I (FOLI, CD19^{+}B220^{+}CD93^{-}CD21^{+}IgM^{+}IgD^{+}), follicular II (FOLII, CD19^{+}B220^{+}CD93^{-}CD21^{+} IgM^{hi}IgD^{+}), marginal zone progenitors (MZP, CD19^{+}B220^{+}CD93^{-}CD21^{hi}IgM^{hi}CD23^{+}), and marginal zone B cells (MZ, CD19^{+}B220^{+}CD93^{-}CD21^{hi}IgM^{hi}CD23^{-}), as previously [1,2]. The data is from (A) 3-10 mice per genotype consolidated from two independent experiments, or (B) 8-11 mice per genotype consolidated from three independent experiments. Bars represent means± SEM; statistical analysis with ANOVA and Dunnett's post-hoc test, comparing each group to the control, * $p<0.05$, ** $p<0.01$, *** $p<0.001$, **** $p<0.0001$, NS - not significant.

Figure S3

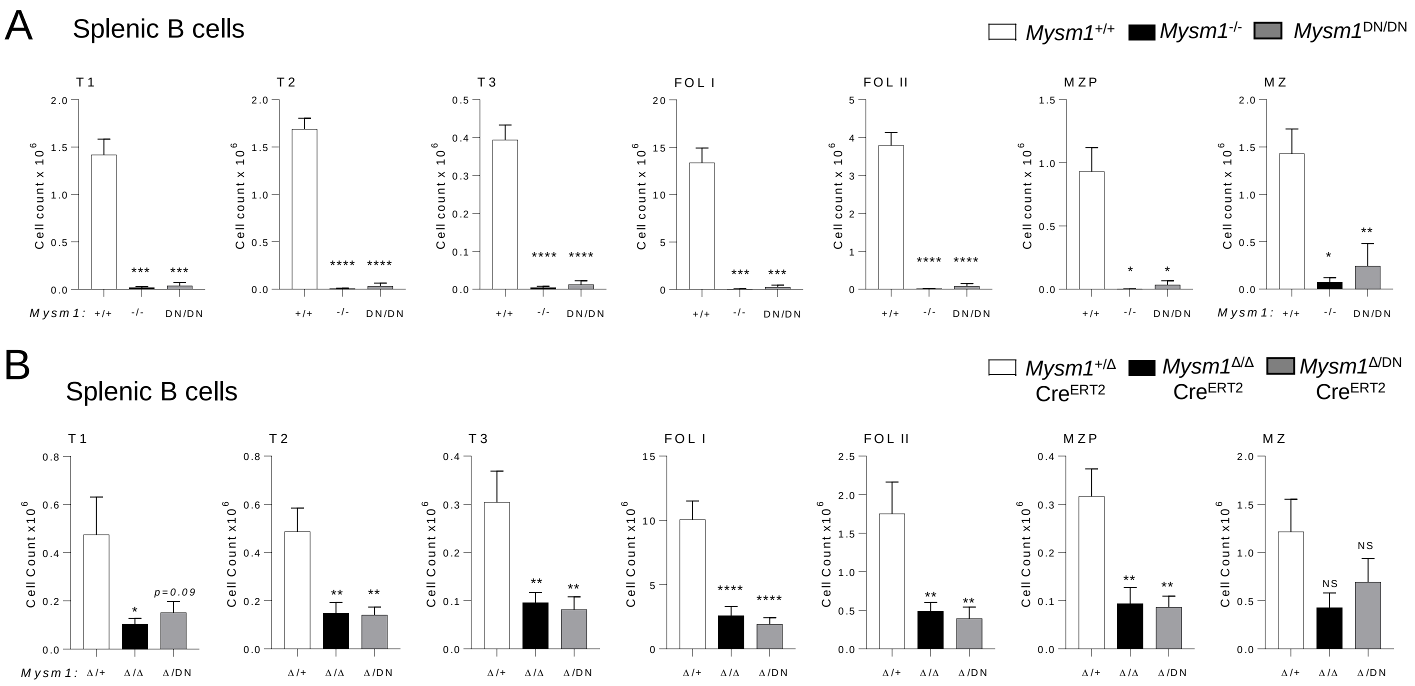
A
Splenic B cells
Mysm1+/+
Mysm1-/-
Mysm1DN/DN
T1
T2
T3
FOL I
FOL II
MZP
MZ
Cell count x 10^6
Mysm1:
+/+
-/-
DN/DN
B
Splenic B cells
Mysm1+/Δ CreERT2
Mysm1Δ/Δ CreERT2
Mysm1Δ/DN CreERT2
Cell Count x10^6
Δ/+
Δ/Δ
Δ/DN
p=0.09
NS

Figure S4. Assessing the cell-intrinsic role of MYSM1 DUB catalytic activity in hematopoiesis and leukocyte development with competitive bone marrow transplantation.

Wild type $CD45.1^+$ bone marrow cells were mixed in a 1:1 ratio with Cre^{ERT2} transgenic bone marrow cells of $Mysm1^{fl/+}$, $Mysm1^{fl/fl}$, or $Mysm1^{fl/DN}$ genotypes, and the mixes were transplanted into three independent cohorts of lethally irradiated wild type $CD45.1^+$ recipient mice. Following full hematopoietic reconstitution by the donor bone marrows, the chimeric mice were administered with tamoxifen to induce the $Mysm1^{fl}$ to $Mysm1^{\Delta}$ allele conversion. The relative contribution of $Mysm1^{\Delta/DN}$, $Mysm1^{\Delta/\Delta}$, and control $Mysm1^{\Delta/+}$ cells to the different hematopoietic and immune cell populations was evaluated by flow cytometry, gating on $CD45.2^+CD45.1^-$ cells. Data is from 3-5 mice per group; bars represent means ± SEM; statistical analysis with ANOVA and Dunnett's post-hoc test comparing each group to the $Mysm1^{\Delta/+}$ control; * $p<0.05$, ** $p<0.01$, *** $p<0.001$, or NS - not significant.

(A) Analysis of the mouse blood, gating on B cells ($B220^+$), CD4 T cells ($CD3^+CD4^+CD8^-$), CD8 T cells ($CD3^+CD8^+CD4^-$), NK cells ($CD3^-NK1.1^+$), monocytes ($CD11b^+Ly6C^{hi}$), and neutrophils ($CD11b^+Ly6G^{hi}$). **(B)** Analyses of B cell precursor cells in the mouse bone marrow, gating on $B220^+$ for all B cells, and on IgM^- $CD43^+CD24^-$ $BP1^-$ for Fraction A, IgM^- $CD43^+CD24^+$ $BP1^-$ for Fraction B, IgM^- $CD43^+CD24^+$ $BP1^+$ for Fraction C, IgM^- IgD^- $CD43^-$ for pre-B cells, IgM^+ IgD^- for immature B cells, and IgM^+ IgD^+ for mature B cells. **(C)** Analyses of T cell development in the thymus, gating on $CD4^-CD8^-$ for double-negative thymocytes (DN), $CD4^+CD8^+$ for double-positive thymocytes (DP), $CD4^+CD8^-$ for CD4 single-positive, and $CD4^-CD8^+$ for CD8 single-positive thymocytes. **(D)** Analysis of hematopoietic stem cells (HSCs) and multipotent progenitors 1-4 (MPP1-4) in mouse bone marrow gated as $Lin^-cKit^+Sca1^+$ followed by $CD150^+CD48^-CD34^-Flt3^-$ for HSCs, $CD150^+CD48^-CD34^+Flt3^-$ for MPP1, $CD150^+CD48^+CD34^+Flt3^-$ for MPP2, $CD150^-CD48^+CD34^+Flt3^-$ for MPP3, and $CD150^-CD48^+CD34^+Flt3^+$ for MPP4 cells. **(E)** Setting the $CD45.1^+$ and $CD45.2^+$ gates for splenic B cells, splenic T cells, and bone marrow neutrophils, using control non-chimeric WT-B6 (CD45.2) and WT-SJL (CD45.1) mice; gates for each of the other cell populations were set independently with the same method.

Figure S4

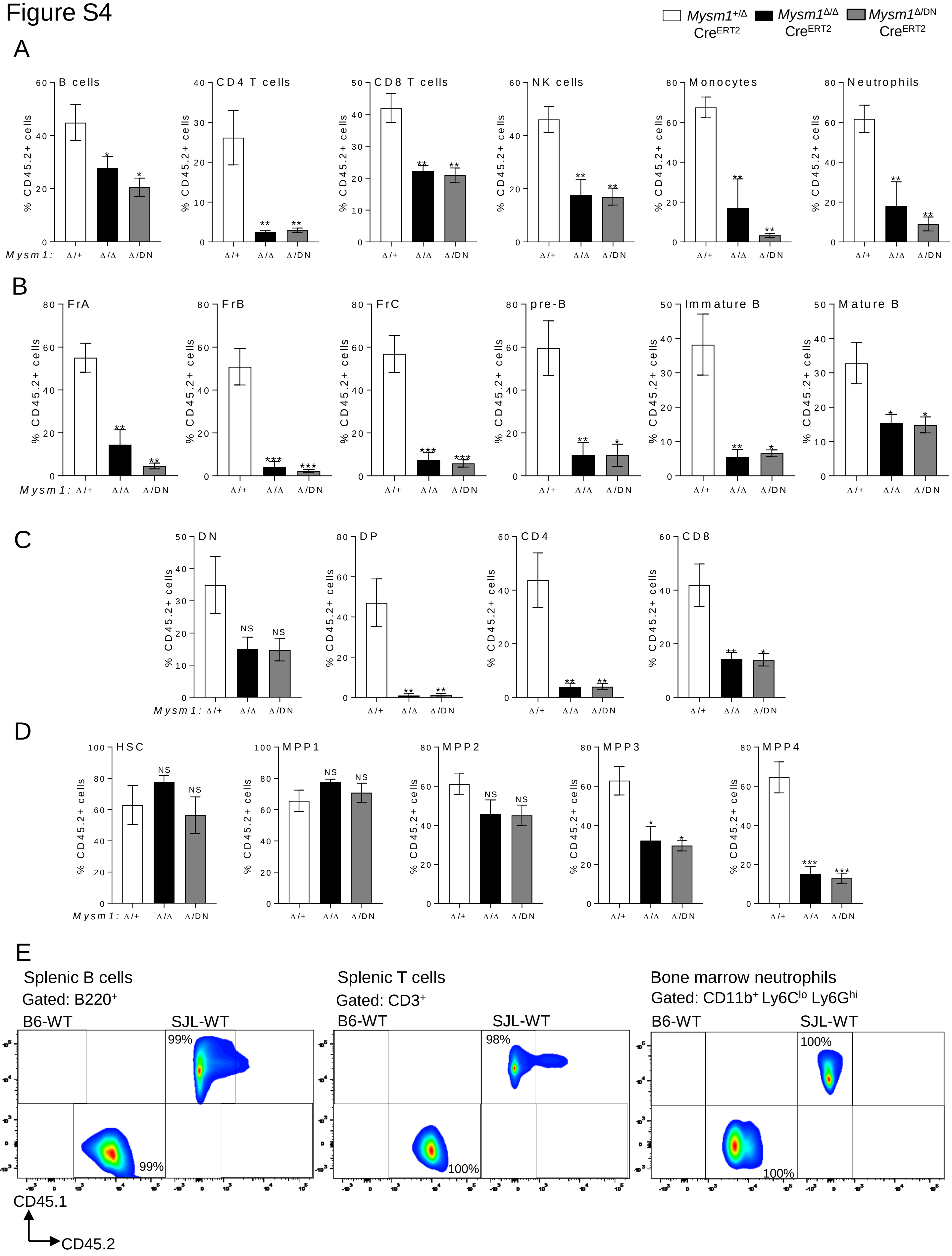

Mysm1+/Δ CreERT2
Mysm1Δ/Δ CreERT2
Mysm1Δ/DN CreERT2
A
B cells
CD4 T cells
CD8 T cells
NK cells
Monocytes
Neutrophils
% CD45.2+ cells
Mysm1: Δ/+ Δ/Δ Δ/DN
B
FrA
FrB
FrC
pre-B
Immature B
Mature B
C
DN
DP
CD4
CD8
D
HSC
MPP1
MPP2
MPP3
MPP4
NS
E
Splenic B cells
Gated: B220+
Splenic T cells
Gated: CD3+
Bone marrow neutrophils
Gated: CD11b+ Ly6Clo Ly6Ghi
B6-WT
SJL-WT
99%
98%
100%
CD45.1
CD45.2

Table S1. Antibodies and Other Reagents Used in Flow Cytometry and Western Blot Analyses

Target	Fluorophore	Product full name	Manufacturer	Catalog number	Clone
A4B7 (LPAM1)	APC	APC anti-mouse LPAM-1 (Integrin α4β7) Antibody	BioLegend	120607	DATK32
B220	APC-eF780	CD45R (B220) Monoclonal Antibody, APC-eFluor™ 780	Invitrogen	47-0452-82	RA3-6B2
B220	Biotin	anti-mouse CD45R/B220 (from Biotin anti-mouse Lineage Panel)	BioLegend	133307 (79752)	RA3-6B2
B220	BV650	Brilliant Violet 650™ anti-mouse/human CD45R/B220 Antibody	BioLegend	103241	RA3-6B2
B220	eFluor450	CD45R (B220) Monoclonal Antibody, eFluor™ 450	eBioscience	48-0452-82	RA3-6B2
B220	PerCP-Cy5.5	PerCP/Cyanine5.5 anti-mouse/human CD45R/B220 Antibody	BioLegend	103236	RA3-6B2
BP1	FITC	CD249 (BP-1) Monoclonal Antibody, FITC	eBioscience	11-5891-82	6C3
CD115	PE-Cy7	PE/Cyanine7 anti-mouse CD115 (CSF-1R) Antibody	BioLegend	135523	AFS98
CD11b	Biotin	anti-mouse CD11b (from Biotin anti-mouse Lineage Panel)	BioLegend	133307 (79749)	M1/70
CD11b	eFluor450	CD11b Monoclonal Antibody, eFluor™ 450	eBioscience	48-0112-82	M1/70
CD11b	PerCP-Cy5.5	PerCP/Cyanine5.5 anti-mouse/human CD11b Antibody	BioLegend	101228	M1/70
CD11c	APC	APC anti-mouse CD11c Antibody	BioLegend	117309	N418
CD11c	BV785	Brilliant Violet 785™ anti-mouse CD11c Antibody	BioLegend	117336	N418
CD11c	BUV737	BUV737 Hamster Anti-Mouse CD11c	BD Biosciences	612796	HL3
CD127 (IL7Ra)	BUV737	BUV737 Rat Anti-Mouse CD127	BD Biosciences	612841	SB/199
CD150	PE-Cy7	PE/Cyanine7 anti-mouse CD150 (SLAM) Antibody	BioLegend	115913	TC15-12F12.2
CD16/CD32	FITC	CD16/CD32 Monoclonal Antibody, FITC	eBioscience	11-0161-82	93
CD172a/SIRPa	PE	PE anti-mouse CD172a (SIRPα) Antibody	BioLegend	144011	P84
CD172a/SIRPa	PerCP-Cy5.5	PerCP/Cyanine5.5 anti-mouse CD172a (SIRPα) Antibody	BioLegend	144009	P84
CD19	PE-Cy7	PE/Cyanine7 anti-mouse CD19 Antibody	BioLegend	115520	6D5
CD19	PerCP-Cy5.5	PerCP/Cyanine5.5 anti-mouse CD19 Antibody	BioLegend	115534	6D5
CD21	APC	APC anti-mouse CD21/CD35 (CR2/CR1) Antibody	BioLegend	123411	7E9
CD23	FITC	CD23 Monoclonal Antibody, FITC	eBioscience	11-0232-85	B3B4
CD24	eFluor450	CD24 Monoclonal Antibody, eFluor™ 450	eBioscience	48-0242-82	M1/69
CD25	FITC	FITC anti-mouse CD25 Antibody	BioLegend	102006	PC61
CD3	APC	APC anti-mouse CD3 Antibody	BioLegend	100235	17A2
CD3	Biotin	anti-mouse CD3e (from Biotin anti-mouse Lineage Panel)	BioLegend	133307 (79751)	145-2C11
CD3	PE	PE anti-mouse CD3 Antibody	BioLegend	100206	17A2
CD3	PerCP-Cy5.5	PerCP/Cyanine5.5 anti-mouse CD3 Antibody	BioLegend	100217	17A2
CD34	BV421	BV421 Rat Anti-Mouse CD34	BD Biosciences	562608	RAM34
CD4	PE-Cy7	Brilliant Violet 785™ anti-mouse CD4 Antibody	BioLegend	100552	RM4-5
CD4	PerCP-Cy5.5	PerCP/Cyanine5.5 anti-mouse CD4 Antibody	BioLegend	116012	RM4-4
CD41	BUV395	BUV395 Rat Anti-Mouse CD41	BD Biosciences	564056	MWReg30
CD43	BUV395	BUV395 Rat Anti-Mouse CD43	BD Biosciences	740224	S7
CD44	APC	CD44 Monoclonal Antibody, APC	eBioscience	17-0441-83	IM7
CD45	BUV395	BUV395 Rat Anti-Mouse CD45	BD Biosciences	564279	30-F11
CD45.1	BUV737	BUV737 Mouse Anti-Mouse CD45.1	BD Biosciences	612811	A20
CD45.2	PE-Cy7	PE/Cyanine7 anti-mouse CD45.2 Antibody	BioLegend	109830	104
CD48	PerCP-Cy5.5	PerCP/Cyanine5.5 anti-mouse CD48 Antibody	BioLegend	103422	HM48-1
CD5	APC-Cy7	CD5 Monoclonal Antibody, PerCP-Cyanine5.5	eBioscience	45-0051-82	53-7.3
CD64 (FcγRI)	PE-Cy7	PE/Cyanine7 anti-mouse CD64 (FcγRI) Antibody	BioLegend	139313	X54-5/7.1
CD8	APC	CD8a Monoclonal Antibody, APC	eBioscience	17-0081-82	53-6.7
CD8	BV650	Brilliant Violet 650™ anti-mouse CD8a Antibody	BioLegend	100742	53-6.7
CD8	eFluor450	CD8a Monoclonal Antibody, eFluor™ 450	eBioscience	48-0081-82	53-6.7
CD8	PerCP-Cy5.5	PerCP/Cyanine5.5 anti-mouse CD8a Antibody	BioLegend	100734	53-6.7
CD93	PerCP-Cy5.5	PerCP/Cyanine5.5 anti-mouse CD93 (AA4.1, early B lineage) Antibody	BioLegend	136512	AA4.1
cKIT	BV650	Brilliant Violet 650™ anti-mouse CD117 (c-kit) Antibody	BioLegend	135125	ACK2
cKIT	eFluor450	CD117 (c-Kit) Monoclonal Antibody, eFluor™ 450	eBioscience	48-1171-82	2B8
CLEC12A	APC	APC anti-mouse CD371 (CLEC12A) Antibody	BioLegend	143406	5D3/CLEC12A
F4/80	BV785	Brilliant Violet 785™ anti-mouse F4/80 Antibody	BioLegend	123141	BM8
FLT3	PE	CD135 (Flt3) Monoclonal Antibody, PE	eBioscience	12-1351-83	A2F10
IgD	APC	APC anti-mouse IgD Antibody	BioLegend	405714	11-26c.2a
IgD	Pacific Blue	Pacific Blue™ anti-mouse IgD Antibody	Biolegend	405711	11-26c.2a
IgM	PE	IgM Monoclonal Antibody, PE	eBioscience	12-5790-83	II/41
Ki67	BV650	Brilliant Violet 650™ anti-mouse/human Ki-67 Antibody	Biolegend	151215	11F6
Ly6C	PE	PE anti-mouse Ly-6C Antibody	BioLegend	128007	HK1.4
Ly6G	APC-Cy7	APC/Cyanine7 anti-mouse Ly-6G Antibody	BioLegend	127623	1A8
Ly6G	Biotin	Biotin anti-mouse Ly-6G Antibody	BioLegend	127603	1A8
Ly6G	PerCP-Cy5.5	PerCP-Cyanine5.5 Anti-Mouse Ly-6G	Tonbo	65-1276-U1000	1A8
MHCII (I-A/I-E)	BV650	Brilliant Violet 650™ anti-mouse I-A/I-E Antibody	BioLegend	107641	M5/114.15.2
NK1.1	FITC	FITC anti-mouse NK-1.1 Antibody	BioLegend	108706	PK136
NK1.1	PerCPCy5.5	PerCP/Cyanine5.5 anti-mouse NK-1.1 Antibody	BioLegend	108727	PK136
p53	AF488	p53 (1C12) Mouse mAb (Alexa Fluor ® 488 Conjugate)	NEB	2015S	1C12
PDCA1	FITC	FITC anti-mouse CD317 (BST2, PDCA-1) Antibody	BioLegend	127008	927
SCA1	APC	Ly-6A/E (Sca-1) Monoclonal Antibody, APC	eBioscience	17-5981-83	D7
SCA1	APC-Cy7	APC/Cyanine7 anti-mouse Ly-6A/E (Sca-1) Antibody	BioLegend	108126	D7
TER119	Biotin	anti-mouse TER-119/Erythroid Cells (from Biotin anti-mouse Lineage Panel)	BioLegend	133307 (79748)	TER-119
TER119	PerCP-Cy5.5	PerCP/Cyanine5.5 anti-mouse TER-119/Erythroid Cells Antibody	BioLegend	116228	TER-119
XCR1	APC-Cy7	APC/Cyanine7 anti-mouse/rat XCR1 Antibody	BioLegend	148224	ZET
VIABILITY	V506	Fixable Viability Dye eFluor™ 506	eBioscience	65-0866-18	
Streptavidin	BV785	Brilliant Violet 785™ Streptavidin	BioLegend	405249	
MYSM1		Recombinant Anti-MYSM1 antibody [EPR18657] (ab193081)	Abcam	ab193081	EPR18657
beta-Actin		beta-Actin (D6A8) Rabbit mAb	NEB	8457S	D6A8
Anti-Rabbit IgG		Rabbit TrueBlot® Anti-Rabbit IgG HRP	VWR (Rockland Inc)	CA89409-752	

Supplemental Table S2. Primers for RT-qPCR analysis of *Mysm1* transcript.

Target	Forward primer	Reverse primer
Mysm1 exon 15-16	GGCTTCTAGGAGGAAGATACTC	GATACAGGATCCATCTCACACT
Mysm1 exon 16-17	CAGTGTGAGATGGATCCTGTAT	CTCCTCTGGAGAAGTAACTCTG
Hprt (housekeeping gene)	CAGGCCAGACTTTGTTGGAT	TTGCGCTCATCTTAGGCTTT

SUPPLEMENTAL REFERENCES

1 Allman, D. & Pillai, S. Peripheral B cell subsets. *Curr Opin Immunol* **20**, 149-157, doi:10.1016/j.coi.2008.03.014 (2008).

2 Lin, Y. H. *et al.* Regulation of B Lymphocyte Development by Histone H2A Deubiquitinase BAP1. *Front Immunol* **12**, 626418, doi:10.3389/fimmu.2021.626418 (2021).
