# Supplementary material for: Deubiquitinase catalytic activity of MYSM1 is essential in vivo for hematopoiesis and immune cell development
Source: Sci Rep. 2023 Jan 7;13:338. doi: 10.1038/s41598-023-27486-7 (PMC9825392; doi:10.1038/s41598-023-27486-7)
Supplement: Supplementary file 2 — Supplementary Information 2. [file 41598_2023_27486_MOESM2_ESM.pdf]

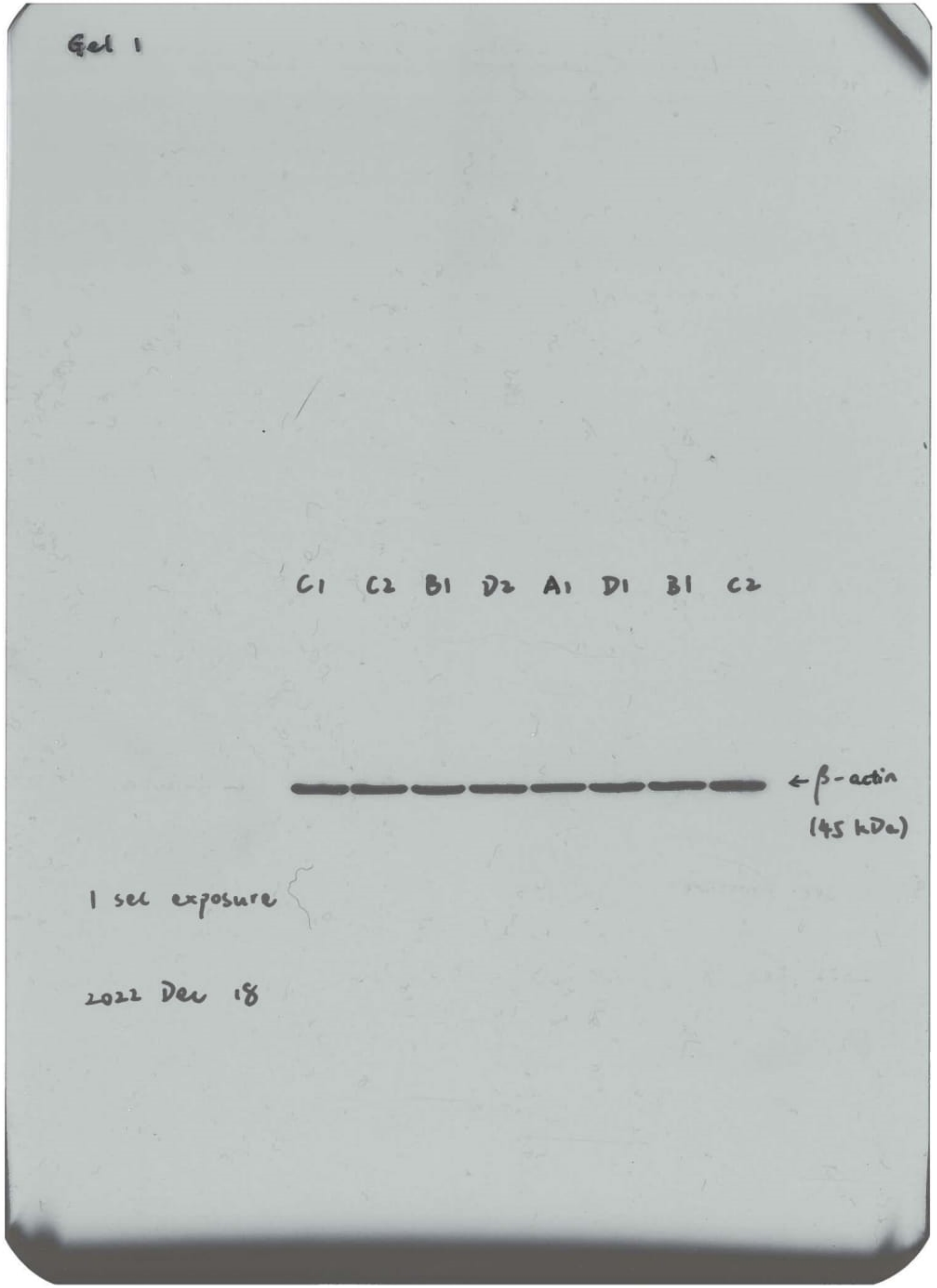

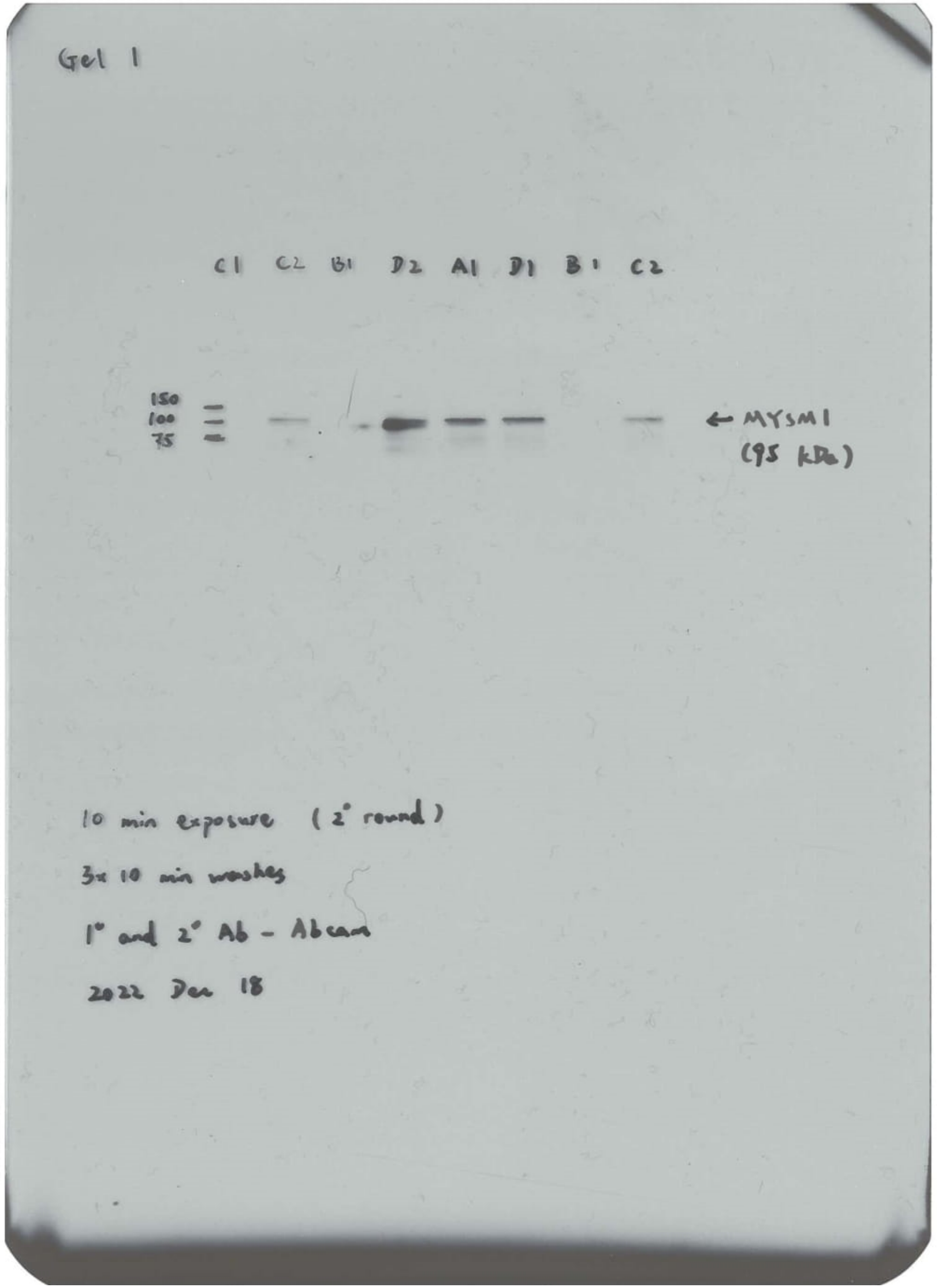

A = MYSM1-DN/DN
B = MYSM1 -/-
C= MYSM1 DN/+
D = MYSM1 +/+
