# Supplementary material for: Deubiquitinase catalytic activity of MYSM1 is essential in vivo for hematopoiesis and immune cell development
Source: Sci Rep. 2023 Jan 7;13:338. doi: 10.1038/s41598-023-27486-7 (PMC9825392; doi:10.1038/s41598-023-27486-7)
Supplement: Supplementary file 3 — Supplementary Information 3. [file 41598_2023_27486_MOESM3_ESM.pdf]

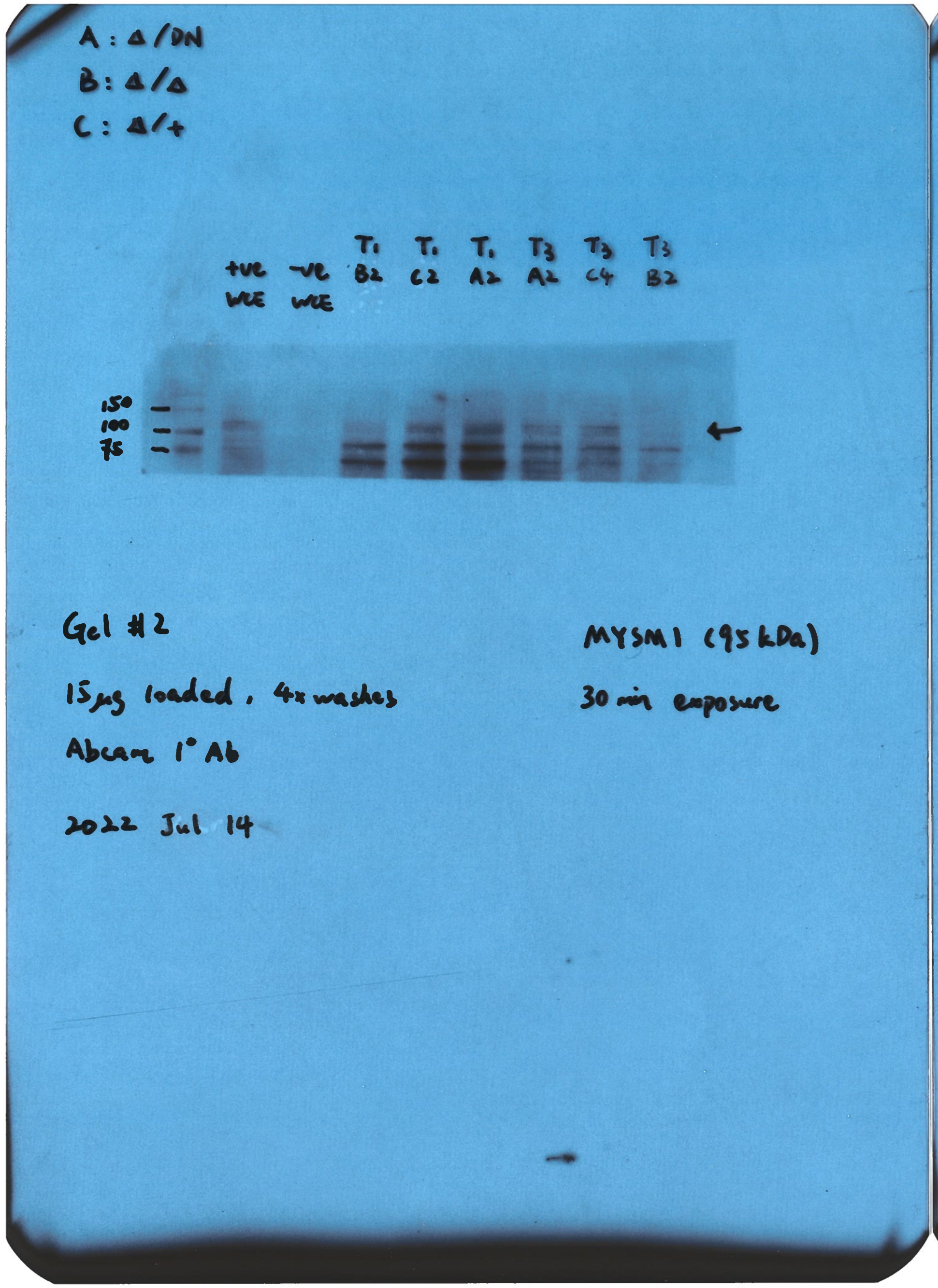
A : Δ/DN
B : Δ/Δ
C : Δ/+
+ve WCE
-ve WCE
T1 B2
T1 C2
T1 A2
T3 A2
T3 C4
T3 B2
150
100
75
Gel #2
15μg loaded, 4x washes
Abcam 1° Ab
2022 Jul 14
MYSM1 (95 kDa)
30 min exposure

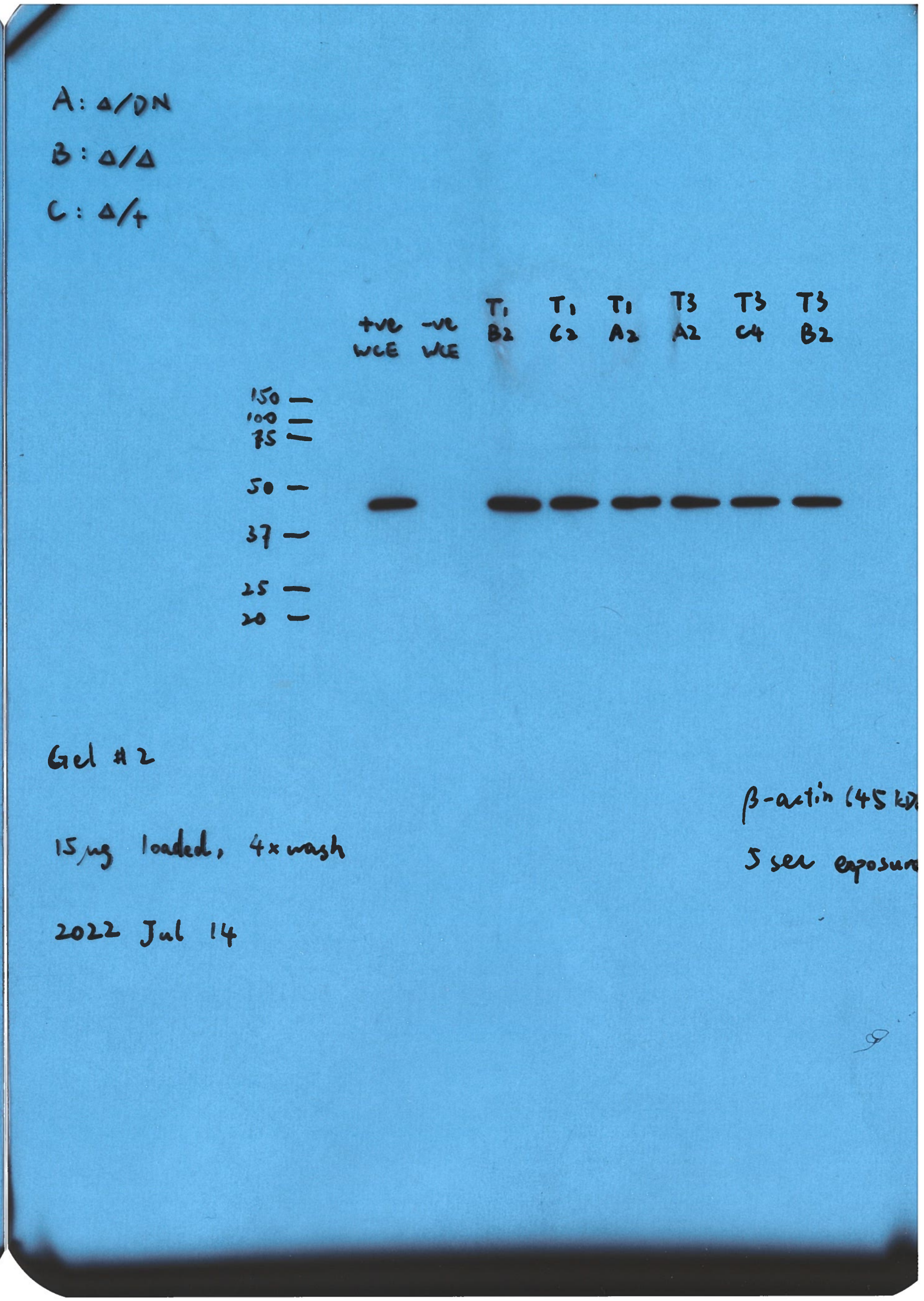
A : Δ/DN
B : Δ/Δ
C : Δ/+
+ve WCE
-ve WCE
T1 B2
T1 C2
T1 A2
T3 A2
T3 C4
T3 B2
150
100
75
50
37
25
20
Gel #2
15μg loaded, 4x wash
2022 Jul 14
β-actin (45 kD
5 sec exposure
